# Dose optimization of an adjuvanted peptide-based personalized neoantigen melanoma vaccine

**DOI:** 10.1371/journal.pcbi.1011247

**Published:** 2024-03-01

**Authors:** Wencel Valega-Mackenzie, Marisabel Rodriguez Messan, Osman N. Yogurtcu, Ujwani Nukala, Zuben E. Sauna, Hong Yang

**Affiliations:** 1 Office of Biostatistics and Pharmacovigilance, Center for Biologics Evaluation and Research, U.S. Food and Drug Administration, Silver Spring, Maryland, United States of America; 2 Office of Therapeutic Products, Center for Biologics Evaluation and Research, U.S. Food and Drug Administration, Silver Spring, Maryland, United States of America; US Army Medical Research and Materiel Command: US Army Medical Research and Development Command, UNITED STATES

## Abstract

The advancements in next-generation sequencing have made it possible to effectively detect somatic mutations, which has led to the development of personalized neoantigen cancer vaccines that are tailored to the unique variants found in a patient’s cancer. These vaccines can provide significant clinical benefit by leveraging the patient’s immune response to eliminate malignant cells. However, determining the optimal vaccine dose for each patient is a challenge due to the heterogeneity of tumors. To address this challenge, we formulate a mathematical dose optimization problem based on a previous mathematical model that encompasses the immune response cascade produced by the vaccine in a patient. We propose an optimization approach to identify the optimal personalized vaccine doses, considering a fixed vaccination schedule, while simultaneously minimizing the overall number of tumor and activated T cells. To validate our approach, we perform *in silico* experiments on six real-world clinical trial patients with advanced melanoma. We compare the results of applying an optimal vaccine dose to those of a suboptimal dose (the dose used in the clinical trial and its deviations). Our simulations reveal that an optimal vaccine regimen of higher initial doses and lower final doses may lead to a reduction in tumor size for certain patients. Our mathematical dose optimization offers a promising approach to determining an optimal vaccine dose for each patient and improving clinical outcomes.

## Author summary

The development of neoantigen cancer vaccines have rapidly increased over the past decade with the advancement of next-generation sequencing technologies to determine immunogenic peptides from patient’s somatic mutations. However, traditional methods to determine the cancer vaccine dose often produce suboptimal clinical outcomes. This work use our previous mathematical model to represent the immunological cascade at the cellular and subcellular levels elicited by the vaccine dose, and focuses on developing a mathematical optimization approach to identify the optimal vaccine dose to minimize two objective functionals, (i) minimize the amount of peptide dose and tumor size, and (ii) minimize the number of activated T cells in addition to the objective functional i. The optimization approach allows the identification of optimal vaccine doses among a set of tested doses for a higher clinical benefit in tumor reduction. To demonstrate and validate our optimization approach, we perform *in silico* experiments on six patients with melanoma from a clinical trial study. The results show that the predicted optimal vaccine doses can provide higher clinical benefit in tumor reduction when compared to the clinical trial doses for some patients.

## Introduction

Cancer is the second-leading cause of death globally, accounting for approximately one in every six deaths in 2018 [1, 2]. Current cancer treatments, including surgery, radiotherapy, chemotherapy, and immunotherapy, can improve a patient’s clinical outcome, but long-term survival is often impacted by the immunosuppressive environment that cancer patients experience [3]. Therapeutic cancer vaccines provide clinical benefits to cancer patients by eliciting an anti-tumor immune response, increasing survival and long-term remission [4–6]. However, selection of optimal dose and regimen for personalized cancer vaccines is one of the challenges in the rapid emerging number of clinical trials due to several factors, including lack of systematic approaches to test different platforms to induce immune responses, small patient sample size to efficiently characterize the shape of the immune response curve, tumor heterogeneity, inadequate study population, and limited quantitative modeling methods that help understand the most suitable dose-response relationships [4, 7, 8].

In recent years, mechanistic and quantitative systems pharmacology (QSP) models have proved to be useful for understanding the complex interactions among the immune system, tumors, and therapeutic interventions [9–13]. These mathematical tools allow for modeling specific cell populations such as dendritic, memory T, helper T, cytotoxic T, or natural killer cells, as well as the tumor microenvironment [14–20], and have been used to better understand and improve multiple cancer treatments/vaccine regimens [21–26], as well as a tool to quantitatively measure immunotherapy responses of certain human immune cell functions such as tumor antigen-specific T cell responses that may lead to tumor reduction [4].

Classical methods for drug assessment during a phase I clinical trial in oncology are the accelerated titration designs, the canonical 3+3 designs, or other similar derived designs which, frequently, produce suboptimal results for patients [27–30]. These dose design methods use a small group of people to determine the dose-limiting toxicity and maximum tolerated dose. However, these methods are likely to provide inadequate estimates when developing personalized drugs [31, 32]. Understanding the patient’s history and the variability of drug responses can potentially improve drug efficacy and mitigate the risk of adverse events.

Due to patient heterogeneity (e.g., rapidly progressive disease, immune suppression, non-immunogenic cancers, and slow immune response) in clinical trials, dosing regimens for individual patients are difficult to test. In general, a vaccination strategy with insufficient amounts of antigen may not be effective, whereas excess doses could present practical constraints and safety concerns, including cytokine syndrome. Thus, an optimal cancer vaccine dose needs to be personalized for each patient [4]. An optimal cancer vaccine dose for each individual patient may be explored using compartmental models involving differential equations; however, not much work has been done in this area.

The goal of this study is to propose a novel approach to quantitatively determining the optimal composition, including peptide and adjuvant, of a personalized cancer vaccine. To achieve this goal, we propose two optimization problems using the immunological model we developed previously [33]. The first optimization problem focuses on minimizing the overall number of tumor cells and total vaccine exposure throughout the treatment. The second problem seeks to identify the minimum number of activated *CD*4^+^ and *CD*8^+^ T cells required for achieving the largest reduction in tumor cell count. While the first problem optimizes for efficacy (reduction of tumor cell count), the second problem optimizes for both efficacy and safety (an excessive immune response may pose a potential safety risk). It is essential to note that this approach may not consistently yield an optimal dose with significantly better clinical outcomes in overall tumor reduction compared to any of the tested doses. The optimal dose may, in some cases, provide clinical outcomes similar to those of other tested doses. We apply both optimization problems to six patients with advanced melanoma [34] to investigate whether these patients could have benefited from an optimal personalized vaccine doses.

## Methodology

### The model

We use our compartmental model published previously [33], which captures the interactions among the human immune system, tumor burden, and a personalized neoantigen peptide cancer vaccine. In this paper, we refer to this model as MRM.

**Fig 1:**
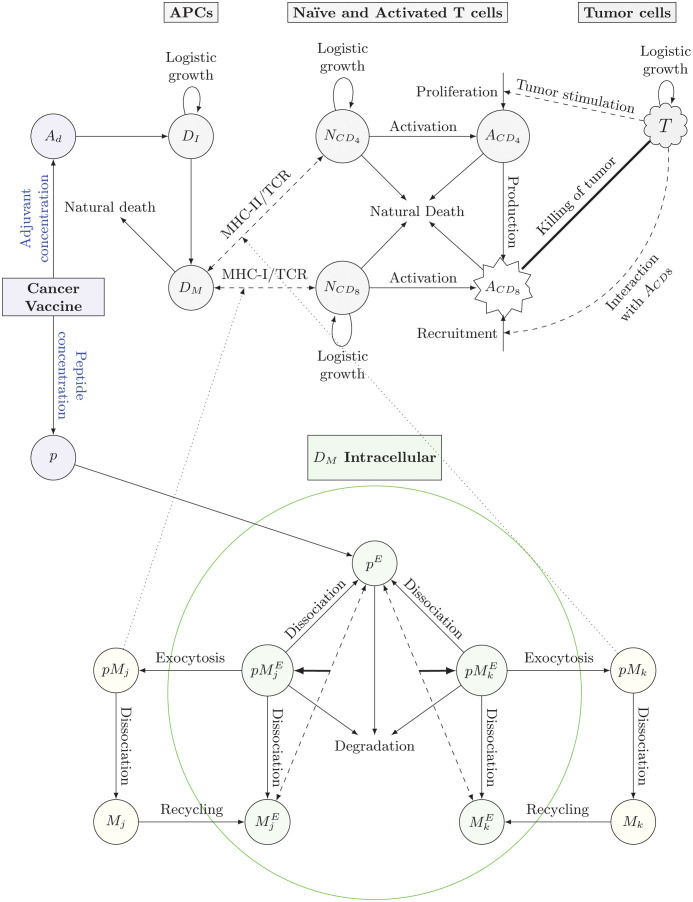
Multiscale flow diagram of immunogenicity to cancer vaccine. The immunological mechanism starts when a patient receives a cancer vaccine, a combination of immunogenic peptides and adjuvant. Adjuvant helps enhance the maturation of immature DCs for antigen presentation at the mature DC surface. Endocytosed peptides interact with MHC-I/II molecules at the subcellular level in matured DCs through binding, dissociating or degradation. Subsequently, antigen-specific T cells are activated by peptides bound to MHC-I/II. Only activated *CD*8^+^ T cells can kill tumor cells. Nonetheless, activated *CD*4^+^ T cells can help activate *CD*8^+^ by tumor stimulation or secretion of IL-2. The solid (dashed) arrows indicate direct (indirect) interactions between the populations at the cellular or subcellular level. The dotted arrows indicated interactions between populations at the subcellular and cellular levels. (APCs: Antigen presenting cells, TCR: T cell receptors. *j* and *k* determine the number of specific MHC-I/II allelic molecules.

The MRM model is deterministic and consists of a set of nonlinear ordinary differential equations (ODEs) with non-negative initial conditions. The model describes the key events associated with an immune reaction to a cancer vaccine at the cellular and subcellular levels, an adjuvanted peptide-based cancer vaccine (see Equations (1)-(2) in S1 Appendix), as well as cell dynamics of the immune system at the molecular (see Equations (5)-(9) in S1 Appendix) and cellular level (see Equations ((3)-(4)) and ((10)-(13) in S1 Appendix). These equations are all interconnected to represent the immune response cascade elicited by a cancer vaccine. At the molecular level, the MRM model focuses on the processing and presentation of neoantigen molecules primarily by dendritic cells (DCs) and captures the subcellular dynamics of endosomal peptides involving major histocompatibility complex (MHC) classes I and II in DCs. At the cellular level, the model presents the evolution of immature and mature DCs, naïve and activated T cells, and tumor cells throughout the course of the treatment with the cancer vaccine. The key immunological processes at the cellular level are activation of DCs by the adjuvant, activation of naïve *CD*4^+^ and *CD*8^+^ T cells by mature DCs carrying peptide-bound (i.e., p-MHC) molecules, proliferation and differentiation of T cells, and elimination of tumor cells by activated *CD*8^+^ T cells. A flow diagram of MRM model is shown in Fig 1. A summary of all model variables with their corresponding definitions and units is shown in Table 1.

**Table 1:** Description of model population (or state) variables including units [33].

	Population	Definition	Units
**Vaccine**	*p*	Peptide Concentration	pmol
	*A*_*d*_	Adjuvant Concentration	mg/L
**Cellular**	*D*_*I*_	Immature DCs	cells
	*D*_*M*_	Mature DCs	cells
	*N*_*CD*4_	Naïve *CD*4^+^ T cells	cells
	*N*_*CD*8_	Naïve *CD*8^+^ T cells	cells
	*A*_*CD*4_	Activated *CD*4^+^ T cells	cells
	*A*_*CD*8_	Activated *CD*8^+^ T cells	cells
	*T*	Tumor cells	cells
**Molecular**	*p* ^*E*^	Endosomal peptide fragments	pmol
	$M_{s}^{E}$	Free Endosomal MHC-I or MHC-II	pmol
	$pM_{s}^{E}$	Endosomal p-MHC-I/II	pmol
	*pM*_*s*_	p-MHC-I/II on mature DC membrane	pmol
	*M*_*s*_	Free MHC-I/II on mature DC membrane	pmol

* Subscript *s* = *j* or *s* = *k* determines the MHC-I or MHC-II molecule, respectively.

The MRM model [33] was calibrated using patient-specific data from six patients with melanoma at different disease stages [34] to estimate key model parameters (see Table A and B in S1 Appendix for a summary of model parameters and estimated values). In our previous study [33], *in silico* experiments (virtual simulations) were performed to investigate the response of each patient to cancer immunotherapy and assess changes in tumor sizes, and global sensitivity analysis was performed to identify and study the behavior of parameters that majorly contribute to the uncertainty of outcomes of interest, such as immune response and tumor cell count.

## Dosing optimization problem

In this study, we formulate two optimization problems associated with the MRM model [33]. We will refer to the MRM model as the state system for the purpose of the optimization setup. Our optimization problem is defined based on the settings and patient information from a published clinical trial [34]. The trial consisted of six patients with stage III (Patients 1, 3, 4, and 5) and stage IV (Patients 2 and 6) melanoma who completed a full series of immunogenic personalized neoantigen cancer vaccines and were followed up for approximately six months. Specifically, the treatment consisted of a series of five priming and two booster vaccinations. Each vaccine dose was formulated with a set of immunizing peptides unique to each patient, admixed with an adjuvant (Polyinosinic-polycytidylic acid, and poly-L-lysine (poly-ICLC)).

In order to formulate the dosing-optimization problem for the cancer vaccine model, we use the optimal control theory of ODEs [35]. This theory has been extensively used in the literature to support informative decisions regarding different biological systems [10, 21, 23, 26, 36, 37]. We followed the three main steps to formulate an optimal control problem: (1) define a biological system (e.g., a system of ODEs), (2) define a set of admissible controls, and (3) define an objective functional or target that entails the purpose of the optimization. Once the optimization problem is defined, we derive a set of necessary conditions that the optimal control as well as the corresponding states must satisfy using Pontryagin’s Maximum Principle [35]. Lastly, we use the necessary conditions to numerically solve the optimization problem using the Forward-Backward Sweep Method. Below we elaborate the process of dosing-optimization formulation step by step.

### The biological system

To formulate our optimal control problem, we use the MRM model [33] with some assumptions on the peptide and adjuvant compartments. The earlier published MRM model assumed that a series of scheduled vaccine doses are administrated to a patient instantaneously at determined time points using a Dirac delta function and where each vaccine dose consisted of fixed concentrations of peptide and adjuvant (see Equations (1)-(2) in S1 Appendix). However, in this study, the goal is to find a set of peptide and adjuvant concentrations that is optimal for the specified target. We assume the functions *Dose*_*p*_(*t*) and *Dose*_*a*_(*t*) respectively, representing the vaccine concentration composed of peptides (pmol) and adjuvant (mg), are piecewise functions of time that take nonzero values during the time of vaccination $\left(\tau_{i},\tau_{i}+\frac{1}{\rho }\right)$, and are zero otherwise. Based on the vaccination schedule described in [34], we assume that the vaccine is given to patients at a fixed schedule *τ*_*i*_ = 0, 3, 7, 14, 21, 83, 139 days for *i* = 1, …, 7. Moreover, *ρ* = 0.001 days^−1^ or 86.4 seconds, which is an approximation of the time it takes for a subcutaneous vaccination process [38].

Based on the above assumptions, our proposed rates of changes of peptide and adjuvant concentrations are described by Eqs (1) and (2)

$$\begin{array}{c} \frac{dp}{dt}=\underset{\text{Peptide}\;\text{Dose}\;\text{Administration}}{\underset{︸}{\rho \cdot {Dose}_{p}\left(t\right)}}-\underset{\text{Endocytosis}\;\text{by}\;\text{DCs}}{\underset{︸}{\alpha_{p}p}} \end{array}$$ (1)

$$\begin{array}{c} \frac{dA_{d}}{dt}=\underset{\text{Adjuvant}\;\text{Dose}\;\text{Administration}}{\underset{︸}{\rho \cdot Dose_{a}\left(Dose_{p}\left(t\right)\right)}}-\underset{\text{Endocytosis}\;\text{by}\;\text{DCs}}{\underset{︸}{\alpha_{d}A_{d}}} \end{array}$$ (2)

where *α*_*p*_ and *α*_*d*_ are rates of DC uptake for the peptide and the adjuvant molecules, respectively. Reflecting the synergy between these two vaccine components, the amount of adjuvant is determined by a fixed adjuvant:peptide ratio and the amount of peptide in mg

$$\begin{array}{c} Dose_{a}\left(Dose_{p}\left(t\right)\right)=\underset{\text{adjuvant:peptide}\;\text{ratio}}{\underset{︸}{r_{a:p}}}\times \underset{\text{peptide}\;\text{dose}\;\text{in}\;\text{mg}}{\underset{︸}{Dose_{p}^{mg}\left(t\right)}} \end{array}$$ (3)

where

$$\begin{array}{c} Dose_{p}^{mg}\left(t\right)=\left(Dose_{p}\left(t\right)\times \text{molecular}\;\text{weight}\right)/10^{6} \end{array}$$ (4)

converts the pmol concentration of peptides to units in mg. With the assumption that the adjuvant amount depends on the amount of peptides, it is always guaranteed that both vaccine components are present in each vaccine dose. The adjuvant, as an immunostimulatory agent, activates the DCs and leads to their maturation. Peptides are trafficked to the endoplasmic reticulum and endosome of mature DCs, interacting with MHC class I and II molecules.

### Set of admissible controls

In this particular case, we assume a state system (MRM model) with the above assumptions and a set of tested peptide concentrations per unit volume:

$$\begin{array}{c} \begin{array}{cc} V≔\{Dose_{p}\in L^{\infty }\left[0,t_{f}\right]: & \;Dose_{p}^{l}\leq Dose_{p}\left(t\right)\leq Dose_{p}^{u}\} \end{array} \end{array}$$

where *t*_*f*_ is the length of time for the treatment. The lower and upper bounds, $Dose_{p}^{l}$ and $Dose_{p}^{u}$, refer to the allowed minimum and maximum concentrations of peptide. It is assumed that the lower bound, $Dose_{p}^{l}$, is positive because negative values are not practically meaningful. The total number of vaccination doses administered to a patient throughout the whole therapy is *τ*; where *τ* = 7 in our case study. The *L*^∞^[0, *t*_*f*_] notation is the space of all bounded functions in the interval [0, *t*_*f*_].

### The objective functionals

We propose the following two objective functionals:

$$\begin{array}{c} J_{1}\left(Dose_{p},T\right)=J_{T}+J_{V} \end{array}$$ (5)

$$\begin{array}{c} J_{2}\left(Dose_{p},T,A_{CD4},A_{CD8}\right)=J_{T}+J_{V}+J_{\text{T-cells}} \end{array}$$ (6)

where

$$J_{T}=\int_{0}^{t_{f}}A_{1}\cdot T\left(t\right)\;dt+A_{1}\cdot T\left(t_{f}\right)$$

includes two terms, the tumor cells over the course of the treatment, $\int_{0}^{t_{f}}T(t)\;dt$, and tumor cells at the final time, i.e., *T*(*t*_*f*_). The integral

$$J_{V}=\int_{0}^{t_{f}}B\cdot Dose_{p}^{2}\left(t\right)\;dt$$

measures the total amount of peptide concentration and

$$J_{\text{T-cells}}=\int_{0}^{t_{f}}A_{2}\cdot A_{CD4}\left(t\right)+A_{2}\cdot A_{CD8}\left(t\right)\;dt$$

represents the total number of activated T cells (*A*_*CD*4_ and *A*_*CD*8_) from the beginning to the end of the therapy. Note that by optimizing the peptide concentration through *J*_*V*_, the vaccine dose (including peptide and adjuvant) is implicitly optimized since we set a fixed ratio for adjuvant:peptide.

Both objective functionals *J*_1_ and *J*_2_ share the terms *J*_*T*_ and *J*_*V*_, but *J*_2_ has an additional term, *J*_T-cell_. This means that both objective functionals target high tumor killing and low vaccine concentrations, but *J*_2_ integral additionally targets minimizing the excess T cell response, which may adversely affect the safety of the treatment. An excessive T cell response could cause autoimmune reactions and tissue damage in patients [39, 40]. Note that the term *J*_*V*_ has a value of 0 except for a short period of time (*τρ*^−1^, assumed to be the average time among patients used for all injections throughout the whole therapy).

Moreover, we establish the total immune and tumor responses by calculating the areas under the curve (AUC) of the time series of activated T cell populations (*A*_*CD*4_ + *A*_*CD*8_), and the tumor cell population (*T*), respectively, which is the same as the value of *J*_*T*_ and *J*_T-cells_ when *A*_1_ and *A*_2_ are both 1.

The constants *A*_1_, *A*_2_ and *B* are weight parameters that have been normalized between 0 and 1. These weights measure the relative importance of each term in the objective functionals. When these weights are closer to 0 or 1, it indicates a low or high level of importance associated with that specific term. For further details, please refer to the sections on weight parameters and sensitivity analysis in S1 Appendix.

### Optimal dosing problem formulation

We illustrate the optimal dosing problem setup using the objective functionals *J*_1_ and *J*_2_. The optimal dosing problem consists of

$$\begin{array}{c} \underset{Dose_{p}\in V}{\text{min}}\;J_{k}\;\text{for}\;k=1,2 \end{array}$$ (7)

subject to the cancer vaccine immunotherapy model, i.e., the state system with the aforementioned assumptions and non-negative initial conditions. Therefore, our optimization problem is a minimization problem.

The goal of the minimization problem when using *J*_1_ is to find an optimal peptide concentration, $Dose_{p}^{*}$ (not necessarily unique), along the entire duration of the treatment so that the tumor cells are minimized. For *J*_2_, the goal is to minimize the activated T cell count in addition to the tumor cells. We derive the necessary conditions of the minimization problem using Pontryagin’s Maximum Principle, which are described in detail in S1 Appendix.

### Forward Backward Sweep Method (FBSM)

We numerically solve the dose optimization problem using the Forward Backward Sweep Method (FBSM). The FBSM is an iterative algorithm to solve optimal control problems. In general terms, the numerical scheme consists of solving two sets of coupled differential equations and using the optimal control characterization to update the new solutions. The method exits the loop once the desired convergence criteria for solutions are achieved. The FBSM has been extensively used to solve optimal control problems involving biological, immunological, and ecological systems (ODEs, Partial Differential Equations, Difference Equations, Delayed Equations, Integro-differential Equations, etc.) [35]. For details on the numerical convergence and stability of the method, see [41].

In particular, we implement the FBSM to numerically find the solution to the dosing-optimization problem. The state system is solved forward in time, while the adjoint system is solved backward in time using initial conditions for the state variables and *transversality condition* for the adjoints. Different ODE solvers, such as ode45 in Matlab or solve_ivp from scipy.integrate library in Python, can be used to implement this routine. Additionally, we use the optimal dosing characterization to update and find an optimal set of peptide and adjuvant concentrations, and check for convergence using the convergence criteria in [41] described in the code. The state and adjoint systems with the optimal dosing characterization can be found in S1 Appendix.

**Fig 2:**
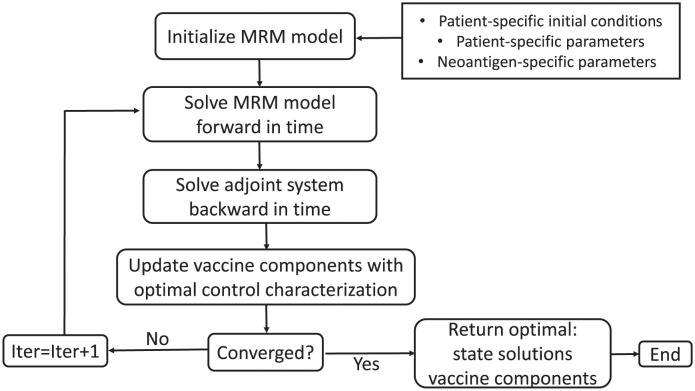
Flow chart of the FBSM. Patient-specific initial conditions are the values of all population variables in Table 1 at the start of the treatment. Patient-specific parameters are T cell recruitment and tumor killing rates, while neoantigen-specific parameters refer to unique immunogenic peptide sequences and binding affinities tailored to each patient. All these values can be found in Tables A and B in S1 Appendix.

The codes to reproduce the results shown in this paper are written in Python and available in the following GitHub repository https://github.com/Wenvalegam/CanVaxDOpt_Model. A flow chart summarizing key steps to apply the FBSM to solve our optimization problem is shown in Fig 2. In the next section, we discuss the dose optimization approach.

### Measuring the performance of an optimal dose

Evaluating the performance of a predicted optimal dose, $Dose_{p}^{*}\in V$, in comparison to any other tested vaccine dose, $Dose_{p}\in V$, requires their corresponding objective functional “scores”. To determine if an optimal vaccine dose is more efficient or not than other tested vaccine doses related to the set V, we use the *J*-ratio

$$\begin{array}{c} \frac{J_{k}\left(Dose_{p}^{*}\right)}{J_{k}\left(Dose_{p}\right)}\;\;\;\text{for}\;k=1\;\text{or}\;2. \end{array}$$ (8)

This *J*-ratio essentially compares the score of an optimal vaccine dose, $J_{k}(Dose_{p}^{*})$, predicted by solving the optimization problem, against any other tested vaccine dose (e.g., the vaccine dose given to patients during the clinical trial and other selected multipliers). Moreover, when the following inequality holds at an optimal vaccine dose

$$J_{k}\left(Dose_{p}^{*}\right)\leq J_{k}\left(Dose_{p}\right)\;\;\;\text{for}\;k=1\;\text{or}\;2,$$

or the *J*-ratio

$$0\leq \frac{J_{k}\left(Dose_{p}^{*}\right)}{J_{k}\left(Dose_{p}\right)}\leq 1\;\;\;\text{for}\;k=1\;\text{or}\;2,$$

the predicted optimal dose is determined to be efficient at achieving the desired goal, which is minimizing either of the objective functionals. Additionally, if the *J*-ratio is close to 1 we can assert the other tested doses perform as well as predicted optimal dose. It is worth noticing that a *J*-ratio close to 1 does not necessarily imply that the predicted optimal dose offers equivalent clinical benefits as any other tested doses. In the next section, we discuss the clinical benefits of a vaccine dose.

However, since there is no guarantee that $J_{k}(Dose_{p}^{*}(t))$ for *k* = 1 or 2 is a global minimum [42], we can also have that for some $Dose_{p}\in V$

$$J_{k}\left(Dose_{p}\right)<J_{k}\left(Dose_{p}^{*}\right)\;\;\;\text{for}\;k=1\;\text{or}\;2.$$

In this case, the *J*-ratio

$$1<\frac{J_{k}\left(Dose_{p}^{*}\right)}{J_{k}\left(Dose_{p}\right)}\;\;\;\text{for}\;k=1\;\text{or}\;2,$$

determines the inefficacy of a predicted $Dose_{p}^{*}$ to minimize an objective functional. When this occurs, $Dose_{p}^{*}$ is not optimal. In the next part, we explain how to overcome this situation.

### Refinement of dose optimization approach

The main issue with a predicted optimal dose, $Dose_{p}^{*}\in V$, is that it could potentially be a local minimizer. To overcome this issue, we propose the following heuristic approach. Let’s assume the *J*-ratio between $Dose_{p}^{*}$ and $\hat{Dose_{p}}\in V$ is greater than 1. We define a sequence of *n*-subsets, decreasing in size, of the set V, $\left\{V^{1},V^{2}\ldots V^{n}\right\}$ with

$$V^{n}\subseteq V^{n-1}\subseteq \cdots \subseteq V^{1}\subseteq V^{0}=V,$$

where the refined set $V^{i}$ is defined as

$$\begin{array}{c} \begin{array}{c} V^{i}≔\{Dose_{p}\in L^{\infty }\left[0,t_{f}\right]:\;Dose_{p}^{l}\leq Dose_{p}\left(t\right)\leq \left(1-\epsilon_{i}\right)\cdot Dose_{p}^{u}\}\subseteq V, \end{array} \end{array}$$

with $\epsilon_{i}\in [0,1-\frac{Dose_{p}^{l}}{Dose_{p}^{u}})$ and *ϵ*_*i*−1_ ≤ *ϵ*_*i*_. The parameter *ϵ*_*i*_ indicates the level of refinement of the set V. We determine a new predicted optimal dose for each refined subset of V, $Dose_{p}^{*,i}\in V^{i}$ for *i* = 1, 2, …, *n*. Hence, a predicted approximation of the optimal peptide dose (global minimizer) is given by

$$\begin{array}{c} Dose_{p}^{*}=\underset{Dose_{p}\in V}{\text{arg}\;\text{min}}\;J_{k}\left(Dose_{p}\right)\approx \underset{i\in \{0,1,\cdots ,n\}}{\text{arg}\;\text{min}}\;J_{k}\left(Dose_{p}^{*,i}\right)\;\text{for}\;k=1\;\text{or}\;2. \end{array}$$ (9)

This nested optimization approach would guarantee that the *J*-ratio between the predicted optimal $Dose_{p}^{*}$ and $\hat{Dose_{p}}$, is always less than or equal to one if the *n*-subsets are systematically selected. To ensure a systematic exploration of the V space, one way to select the *n*-subsets is by iteratively reducing the upper bounds by a fixed amount, as we did in this work. Note that all model and weight parameters are kept the same as initially selected throughout the iteration process.

While our heuristic global optimization approach has the potential to approximate the global minimum, it is important to note that it is not the only approach. Other global optimization techniques, such as those discussed in [42–45], could also be applied within this context.

## Clinical benefits of an optimal vaccine dose

In this section, we illustrate the clinical implications of optimizing the vaccine dose using our framework. We aim to establish a meaningful connection between the abstract weight parameters (*A*_1_, *A*_2_, and *B*) and their clinical interpretations in the context of tumor reduction, immune response enhancement, and vaccine impact.

As previously defined, the weight parameters (*A*_1_, *A*_2_ and *B*) measure the relative importance of each term (*J*_*T*_, *J*_T-cell_ and *J*_*V*_) in our objective functionals. However, this definition of the weights is very abstract if we want to obtain a clinical interpretation of our optimization results. To make these weights clinically relevant, we say that these weights represent the relative importance of minimizing either the tumor, immune, or vaccine terms in our objective functionals at the patient level. Specifically, values ranging from 0 to 1 indicate the low-to-high priority assigned by the patient to each term in the objective functional for the individual patient.

Once optimization returns an optimal vaccine dose, the next question arises: how do we determine whether the predicted optimal vaccine dose offers a clinical benefit to a particular patient? Only checking that the *J*-ratio is less than 1 is not enough to show that there is an actual clinical benefit. For this reason, in addition to finding the *J*-ratio, we introduce the individual ratios from *J*_*T*_ and *J*_T-cell_ in the objective functionals. Thus, we define the following clinical benefit criteria for the optimal vaccine dose.

1. The predicted optimal vaccine dose has a greater clinical benefit in reducing the total number of tumor cells over the course of the treatment than any other tested vaccine dose according to the level of preference selected by the patient if the *J*_*T*_-ratio satisfies the following condition
  $$\begin{array}{c} \frac{J_{T}\left(Dose_{p}^{*}\right)}{J_{T}\left(Dose_{p}\right)}\leq 1. \end{array}$$ (10)
2. The predicted optimal vaccine dose has a greater clinical benefit in reducing the total number of activated T cells along the duration of the therapy than any other vaccine dose according to the selected level of preference chosen by the patient if the *J*_T-cell_-ratio satisfies the following condition
  $$\begin{array}{c} \frac{J_{\text{T-cell}}\left(Dose_{p}^{*}\right)}{J_{\text{T-cell}}\left(Dose_{p}\right)}\leq 1. \end{array}$$ (11)

For both *J*_*T*_ and *J*_T-cell_ ratios, the further these ratios deviate from unity, the more or less clinical benefit an optimal vaccine dose offers when compared to other tested vaccine dose. We do not associate clinical relevance to a *J*_*V*_-ratio since this quantity reflects the impact of a total vaccine dose in a multiplicative manner. Hence, a ratio for *J*_*V*_ that is greater than 1 does not necessarily imply that the total dose of an optimal vaccine is necessarily higher than any other tested vaccine dose.

In particular, to determine the vaccine dose that a patient should receive on a vaccination day, we compute the following integral

$$\begin{array}{c} \begin{array}{cc} \text{Peptide}\;\text{dose} & ≔\int_{\tau_{i}}^{\tau_{i}+\frac{1}{\rho }}\rho \cdot Dose_{p}\left(t\right)\;dt \end{array} \end{array}$$

where *ρ*^−1^ is the average time of injection and *τ*_*i*_ for *i* = 1, 2, …, 7 corresponding to a vaccination day, and thus, the number of doses a patient will receive. The total dose of peptide administered over the whole treatment is computed with the following integral

$$\begin{array}{c} \begin{array}{cc} D(Dose_{p}) & ≔\int_{0}^{t_{f}}\rho \cdot Dose_{p}\left(t\right)\;dt=\sum_{i=1}^{7}\int_{\tau_{i}}^{\tau_{i}+\frac{1}{\rho }}\rho \cdot Dose_{p}\left(t\right)\;dt \end{array} \end{array}$$ (12)

which constitutes the contribution of peptides from each vaccination day. As noted earlier, we assume *Dose*_*p*_(*t*) is 0 outside the vaccine administration periods, $\left[\tau_{i},\tau_{i}+\frac{1}{\rho }\right]$ for *i* = 1, 2, …, 7, to derive the formula in Eq (12). We say that cumulatively the optimal vaccine dose was lower than any other vaccine dose if the *D*-ratio is less than one.

Later in our case study, we will report the *J*, *J*_*T*_, *J*_T-cell_ and *D* ratios to illustrate the clinical benefits of using an optimal vaccine dose.

### Selecting the most clinically effective peptide dose

Our focus now shifts to explaining why the predicted optimal dose in Eq (9) may not always offer sufficient clinical benefits when compared to other tested doses and how to address this challenge.

**Fig 3:**
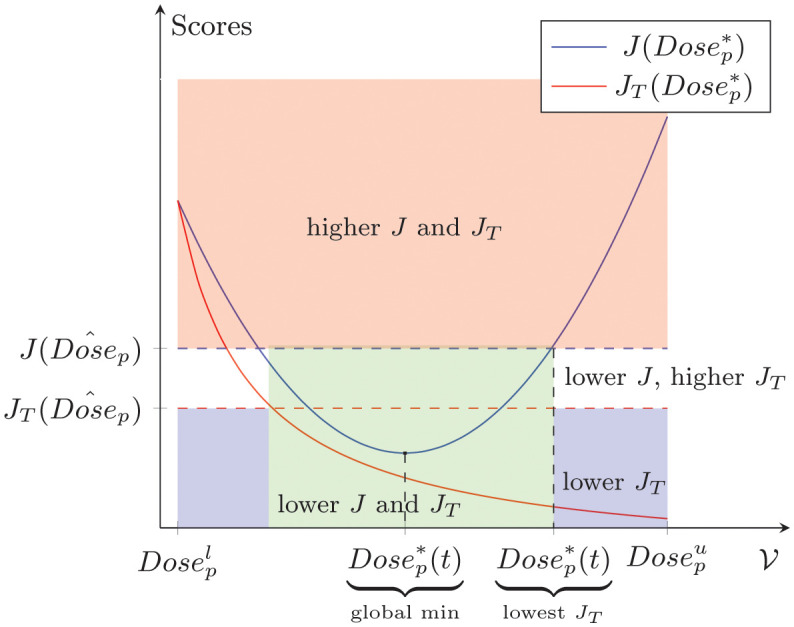
Optimal peptide dose selection. Selecting the predicted optimal peptide dose with higher clinical benefit in overall tumor reduction than the tested vaccine dose. Blue and Red curves correspond to *J* and *J*_*T*_ scores as a function of the predicted optimal doses, $\left\{Dose_{p}^{*,i}\right\}$. Horizontal lines indicate the *J* and *J*_*T*_ scores of tested dose, $\hat{Dose_{p}}$. Red region includes suboptimal doses. Blue region offers doses with potential clinical benefit. Doses below the blue dashed line are optimal with uncertain clinical benefit. Doses between dashed lines are optimal with lower clinical benefit. Doses in the green region are optimal, with a higher benefit in tumor reduction.

The numerical approach outlined in Refinement of dose optimization approach has the potential to identify a set of optimal peptide doses. From this set, we select a global minimizer, $Dose_{p}^{*}$, for the objective functionals. However, it is possible that when computing the *J*-ratio of the global minimizer and other tested dose, $\hat{Dose_{p}}$, the overall reduction of tumor cells is not minimized, that is, the *J*_*T*_ ratio is strictly greater than 1. We illustrate this situation in Fig 3.

In this case, the other tested dose has the potential to have more clinical benefit in overall tumor reduction than the predicted optimal dose. Consequently, we should consider switching our predicted optimal dose in Eq (9) such that it gives us a greater possible tumor reduction when compared to other tested doses.

A predicted optimal peptide vaccine dose should be one that, when compared to any other vaccine dose, provides us with a smaller number of tumor cells over the treatment period. Within our set of predicted optimal peptide doses, $\left\{Dose_{p}^{*,i}\right\}$ for *i* = 1, 2, …, *n*, we identify, $Dose_{p}^{*}$, as optimal and with more overall tumor reduction with respect to other tested vaccine dose, $\hat{Dose_{p}}$, if $Dose_{p}^{*}$ satisfies the following two conditions

$$\begin{array}{c} Dose_{p}^{*}=\underset{i\in \{0,1,\ldots ,n\}}{\text{arg}\;\text{min}}\;\frac{J_{T}\left(Dose_{p}^{*,i}\right)}{J_{T}\left(\hat{Dose_{p}}\right)} \end{array}$$ (13)

and

$$\begin{array}{c} \underset{Dose_{p}^{*}}{\text{min}}\;\frac{J_{k}\left(Dose_{p}^{*}\right)}{J_{k}\left(\hat{Dose_{p}}\right)}\leq 1\;\;\;\text{for}\;k=1\;\text{or}\;2. \end{array}$$ (14)

In Fig 3, we depict an illustrative example of the selection process for a $Dose_{p}^{*}$ with the lowest *J*_*T*_ ratio. This selection process allows us to choose a $Dose_{p}^{*}$ that offers greater clinical benefit in tumor reduction than the other tested vaccine dose.

## Results

### Dosing optimization for six patients with melanoma

In practice, it is very difficult to optimize the dose of a cancer vaccine for individual patients. A dose is typically determined and given to all patients, but some questions remain. For instance: Is the dose provided to an individual patient optimal? Is a fixed dose for all vaccinations warranted, or should the doses for vaccinations over the course of the treatment vary? Our dosing optimization problem is designed to understand the effects produced by a personalized neoantigen cancer vaccine in six patients with melanoma when the concentrations of peptide and adjuvant are varied.

First, we formulate a minimization problem to quantify the impact of the components of the cancer vaccine, namely, peptide and adjuvant (in a fixed ratio), on the total number of tumor cells (*T*) of each patient. With this optimization exercise, we can understand how effective the vaccine is on each patient by predicting tumor size reductions through model simulation. A second optimization problem evaluates the effect of vaccine dose on the total number of tumor cells and on activated T cells (*A*_*CD*4_ + *A*_*CD*8_). In this case, the vaccine dose is optimized by achieving the minimum number of tumor cells (effectiveness) and activated T cells (safety), identifying a vaccine dose that elicits an immune response strong enough to minimize the tumor size, but elicits the minimal number of *T* cells to do so. The latter allays safety concerns. Such a vaccine dose would have the optimal benefit-risk profile.

To solve the optimization problem, we initialize the MRM model (or state system) using the set of parameters described previously [33] and are summarized in Tables A and B in S1 Appendix. The model’s non-specific patient parameters are taken from Table S1 in [33] and shown in Table A in S1 Appendix. Note that the maximum activated T cell recruitment rates (for *A*_*CD*4_ and *A*_*CD*8_), *c*_4_ and *c*; maximum lysis rate by activated T cells, *d*; the dependence of lysis rate between T cells and tumor, λ; and the initial tumor size, *T*(0), are all patient-specific parameters. The values for these parameters are obtained from Table 1 in [33] and shown in Table B in S1 Appendix, where authors used a global optimization tool to find parameters’ best fit to individual patient’s data (with adjusted *R*^2^ between 0.75 and 0.95). Moreover, the off rate of peptide-MHC type I/II with allele *s*, *k*_off,*s*_ for *s* = *j*, *k*, are neoantigen-specific parameters, which can be accessed in the following GitHub repository https://github.com/Wenvalegam/CanVaxDOpt_Model.

The concentrations of neoantigen peptides used in the clinical trial for each patient are presented in Table 2. For more details on how these values are derived, see the supplemental information in [33]. In the clinical trial, each peptide pool (four pools per vaccine) was admixed with 0.5 mg of Poly-ICLC adjuvant in a volume of 1 ml of aqueous solution. The adjuvant is added to the vaccine formulation to enhance immunogenicity [46]. For instance, on each vaccination day, Patient 1, received a cancer vaccine dose including 3.9 mg of peptides and 2 mg of adjuvant (poly-ICLC) in a volume of 4 ml aqueous solution [34]. The adjuvant:peptide ratio, *r*_*a*:*p*_, used for each patient in the clinical trial can be found in Table 2, which is also used as a fixed ratio in our model simulations for each patient.

**Table 2:** Neoantigen Vaccine dose. Peptide dose and adjuvant:peptide ratio converted from clinical trial data for patients with melanoma [33].

Patient	No. of peptides	Weight (mg)	$Dose_{p}^{\text{pt}}$ (pmol)	Adj:Pep (*r*_*a*:*p*_)
1	13	3.9	119,340	$\frac{2}{3.9}$
2	17	5.1	120,030	$\frac{2}{5.1}$
3	14	4.2	109,570	$\frac{2}{4.2}$
4	14	4.2	116,570	$\frac{2}{4.2}$
5	20	6	110,860	$\frac{2}{6}$
6	20	6	111,820	$\frac{2}{6}$

We assume that the average duration of the clinical trial, including post-vaccination follow-up, is 200 days. The set of tested peptide concentrations per 4 ml of aqueous solution is determined by

$$V≔\{Dose_{p}\in L^{\infty }\left[0,200\right]:\;0.1\times Dose_{p}^{\text{Pt}}\leq Dose_{p}\left(t\right)\leq 3\times Dose_{p}^{\text{Pt}}\}.$$

We also assume that the nested subsets $V^{i}$ for *i* = 1, 2, …10 of V are

$$V^{i}≔\{Dose_{p}\in L^{\infty }\left[0,200\right]:\;0.1\times Dose_{p}^{\text{Pt}}\leq Dose_{p}\left(t\right)\leq \left(3-0.25\cdot i\right)\times Dose_{p}^{\text{Pt}}\}.$$

**Fig 4:**
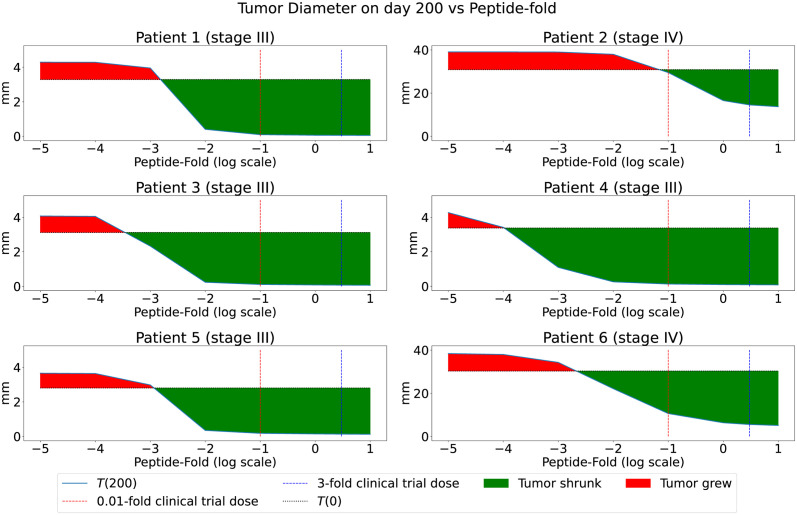
Tumor diameter on day 200 as function of constant peptide concentration on each vaccination day. Vertical lines correspond to the lower and upper bounds set for this dose optimization study for the amount of peptides as the log-folds of the clinical trial dose. The red and green areas indicate tumor growth and reduction from the initial tumor size, respectively.

The $Dose_{p}^{\text{Pt}}$ is the peptide dose used in the clinical trial for each patient and is presented in the 4^th^ column of Table 2. We vary the peptide concentrations from 0.1-fold to up to 3-fold of the $Dose_{p}^{\text{Pt}}$ in our model simulations. These upper and lower bounds of peptide concentrations were chosen to cover the dose range with tumor reduction as demonstrated by the green areas on Fig 4. We also assume that the vaccine dose is safe for each patient within the set V and that there are no significant toxicities. Note that there is no need to select the same lower and upper fold-bounds of peptide concentrations for all the patients, but here we adopt this approach for numerical simplicity. The exact value for the adjuvant dose can be found using Eq (3) with the last column in Table 2 and the corresponding *Dose*_*p*_(*t*) in mg.

Moreover, we choose weights for *J*_1_ to indicate a relative higher level of preference to minimize the total number of tumor cells, but a lower level in minimizing the total vaccine dose, that is, *A*_1_ = 1 and *B* = 1/3. In the case of *J*_2_, we select weights to indicate an equal preference for minimizing the overall tumor, immune responses and the total vaccine dose, that is, *A*_1_ = 1, *A*_2_ = 1 and *B* = 1. The normalization values for the weight parameters can be found in Table C in S1 Appendix.

We apply the dosing-optimization problem to each of the six patients within their set of tested peptide doses (V) to predict outcomes of using an optimal vaccine dose and other suboptimal doses, including the dose used in the clinical trial and their 0.1–3 folds deviations. The solutions to the vaccine dosing minimization problem provide us with a set of optimal concentrations of peptides denoted by $\left\{Dose_{p}^{*,i}(t)\right\}$ for *i* = 0, 1, 2, …, 10 to minimize either *J*_1_ or *J*_2_ objectives according to our weight choices. We select the optimal peptide dose, $Dose_{p}^{*}$, that offers the same or higher clinical benefit in overall tumor reduction compared to the vaccine dose using the two conditions in (13) and (14).

### Vaccine doses

**Fig 5:**
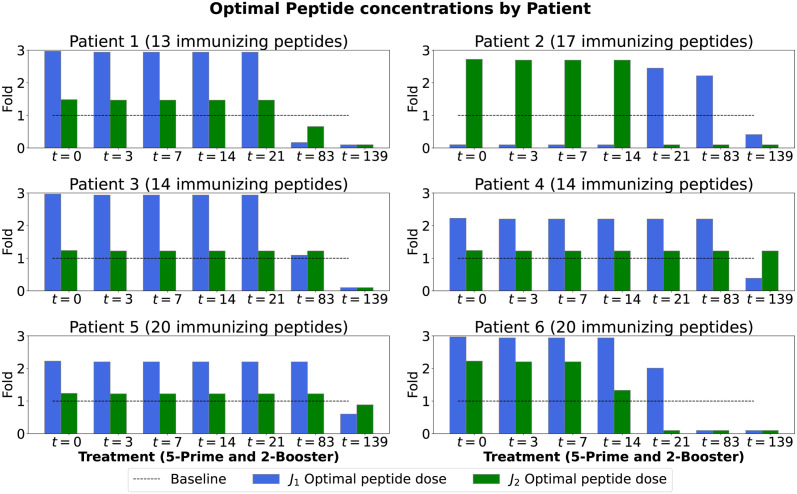
Optimal peptide concentrations. Bar plots correspond to optimal peptide doses as the number of folds of the clinical trial dose for each vaccination using *J*_1_ (blue) and *J*_2_ (green). The horizontal dashed line represents the dose used in the clinical trial (baseline) given in Table 2 for each patient. The total number of immunizing peptides is also reported at the top of each panel. The weights for *J*_1_ are *A*_1_ = 1 and *B* = 1/3, while for *J*_2_ the weights are *A*_1_ = 1, *A*_2_ = 1 and *B* = 1.

**Fig 6:**
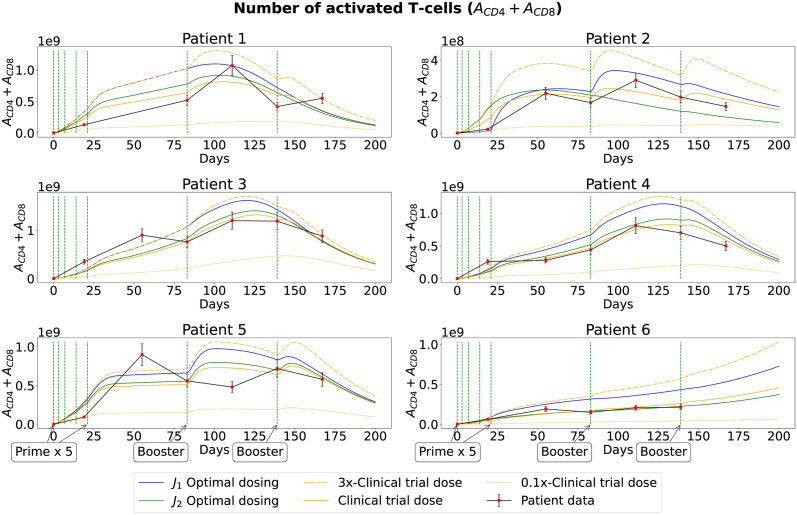
Number of activated T cells. Activated T-cells (*A*_*CD*4_ + *A*_*CD*8_) when optimal (blue and green solid curves, respectively) and suboptimal (dashed, solid and dotted orange are 3, 1, 0.1 folds of clinical trial dose, respectively) vaccine doses are applied to each patient using *J*_1_ and *J*_2_. Red dots represent patients’ measurements at specific times in the clinical trial with 15% standard error. The vertical green dashed lines correspond to the days of vaccination.

**Fig 7:**
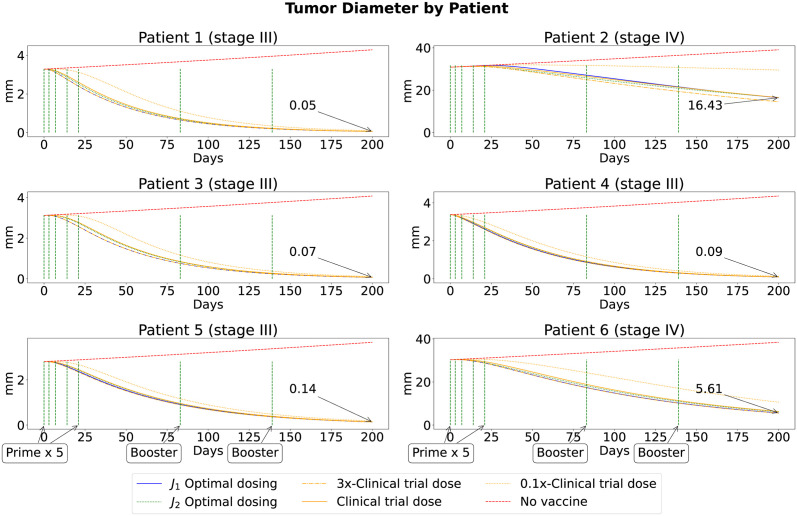
Tumor response. Tumor diameter when optimizing *J*_1_ and *J*_2_, and optimal (blue and green curves), suboptimal (dotted, solid, and dashed orange are 3, 1, and 0.1 folds of clinical trial dose, respectively) and no (red) vaccinations are applied. Numbers with an arrow are pointing at the blue curve when *t* = 200 days, represent the tumor diameter at the end of the treatment (from *J*_1_). The vertical green dashed lines correspond to the days of vaccination.

The optimal dose concentrations of peptide for each vaccination of six patients identified through our simulations are shown in Fig 5 (for *J*_1_ and *J*_2_). These concentrations were used to obtain the optimal immune responses as depicted in Fig 6 and the optimal tumor responses depicted in Fig 7 (blue and green curves for *J*_1_ and *J*_2_, respectively). The exact values for these doses can be found on Tables E-P in S1 Appendix.

The optimal concentrations of peptides when considering minimizing the tumor response only (*J*_1_) are shown as blue bars in Fig 5. For patients 1, 3, and 6 the optimal doses of peptide were 3-times the clinical trial dose (the upper bound of the prespecified range). For Patient 2, low doses for the first four and last vaccinations, and high doses for the other vaccinations are optimal. This result for Patient 2 implies that there may be differences among patients regarding the timing of the higher vaccine doses during the vaccine schedule. With the optimal dose obtained for *J*_1_, all patients achieve tumor size reduction greater or similar to those reported in the clinical trial [34] and are consistent with model predictions [33].

The optimal concentrations of peptides when considering minimizing the T cell and tumor responses (*J*_2_) for each patient are shown as green bars in Fig 5. Interestingly, our optimal peptide dose for Patients 1, 3, 4 and 5 almost exactly matches their clinical trial dose. Patients 2 and 6 require higher doses for the initial vaccinations, but lower doses subsequently. This observation suggests that when trying to minimize the immune response in addition to the tumor response (*J*_2_), for patients (2 and 6) with stage IV melanoma, the initial vaccination dose should be higher and then lowered.

### Immune response

Using the MRM model [33] with our optimization problem, we show the predictions of the number of activated T cells (*A*_*CD*4_ + *A*_*CD*8_) over a 200-day period for six patients with the predicted optimal vaccine doses (for *J*_1_ and *J*_2_), clinical trial dose, and 0.1x and 3x the dose used in the clinical trial.

In Fig 6, we can see that, for all patients, the optimal vaccine dose from *J*_1_ produced a stronger immune response (higher cell count of *A*_*CD*4_ + *A*_*CD*8_ shown by the blue curve) than any other tested vaccine dose. On the other hand, we observe that the immune response with an optimal vaccine dose from *J*_2_, was significantly lower (lower cell count of *A*_*CD*4_ + *A*_*CD*8_ shown by the green curves) than their immune response to clinical trial dose for Patients 2 and 6. These observations demonstrate the importance of establishing objective functionals, which are used to select an optimal vaccine dose for an individual patient.

### Tumor response

In Fig 7, we depict the evolution of the tumor size in mm over 200 days for each patient under optimal and suboptimal vaccine dosing. Note that we first obtained the number of tumor cells and then converted the cell number into mm by using the diameter formula derived in [33]

$$d\left(t\right)=2\cdot {\left(\frac{3\cdot T(t)}{\left(4\pi \cdot 0.7405\cdot 10^{5}\right)}\right)}^{1/3}.$$

In general, we observe that the optimal vaccine dose (blue and green curves) performed slightly better than suboptimal vaccines within the vaccination period (day 0 and 139) of the immunotherapy for all patients, except for Patient 2. However, at the late stage (after day 139), all tested vaccine doses show similar effectiveness in reducing the tumor size for all the patients, with the optimal vaccine doses performing noticeably better for Patient 6. Moreover, when optimizing *J*_2_, Patients 2 and 6 have a larger tumor size on day 200 (16.8 mm and 6.39 mm, respectively).

### Clinical benefits of the optimal vaccine dose

In this section, we summarize the results of optimizing the cancer vaccine dose with respect to *J*_1_ and *J*_2_, and illustrate the clinical benefits (higher tumor reduction or lower T cell activation) they offer when compared to the clinical trial dose. Although the *J*_1_ and *J*_2_ ratios are valuable tools for our optimization approach, they are not enough to offer a clinical interpretation obtained by an optimal vaccine dose. As a result, we must additionally compute the individual *J*_*T*_ and *J*_T-cell_ ratios. These individualized ratios quantify the clinical benefits offered by our predicted cancer vaccine dose when compared to other vaccine doses over the treatment period as we discussed earlier in Clinical benefits of an optimal vaccine dose.

The following tables provide a detailed analysis of the clinical benefits offered by the predicted optimal vaccine doses using the overall and individual ratios for *J*_1_, *J*_2_ and *D*. These tables present a comparative view of the overall tumor reduction, immune response, and vaccine doses.

In the case of *J*_1_ from Table 3, we notice that our predicted optimal dose offers a higher tumor reduction among almost all the patients when compared to the clinical trial dose. However, the total optimal peptide dose was over twice the total clinical trial dose for Patients 1, 3, 5 and 6. It is important to note that the overall optimal vaccine dose is assumed to be safe for Patients 1, 3, 5 and 6.

**Table 3:** Clinical response using *J*_1_. Optimal vaccine dose performance using overall and individual ratios for *J*_1_ together with total vaccine dose ratio when compared to clinical trial dose. Green cells indicate more clinical benefit or a lower total vaccine dose. Red cells indicate less clinical benefit or a higher total vaccine dose.

Pt ID	$\frac{J_{T}(Dose_{p}^{*})}{J_{T}(Dose_{p}^{\text{Pt}})}$	$\frac{D(Dose_{p}^{*})}{D(Dose_{p}^{\text{Pt}})}$	$\frac{J_{1}(Dose_{p}^{*})}{J_{1}(Dose_{p}^{\text{Pt}})}$
1	0.85	2.18	1
2	1	0.8	1
3	0.84	2.32	1
4	0.93	1.98	1
5	0.92	2.02	1
6	0.88	2.04	1

From Table 4 with respect to *J*_2_, our predicted optimal vaccine dose offers slightly better overall tumor reductions and immune responses than the clinical trial dose for Patients 1, 3, 4, and 5. Although for Patients 2 and 6, the optimal vaccine dose can offer more clinical benefits in terms of overall tumor reduction and reducing the total immune response.

**Table 4:** Clinical response using *J*_2_. Optimal vaccine dose performance using overall and individual ratios for *J*_2_ together with total vaccine dose ratio when compared to clinical trial dose. Green cells indicate more clinical benefit or a lower total vaccine dose. Red cells indicate less clinical benefit or a higher total vaccine dose.

Pt ID	$\frac{J_{T}(Dose_{p}^{*})}{J_{T}(Dose_{p}^{\text{Pt}})}$	$\frac{J_{\text{T-cell}}(Dose_{p}^{*})}{J_{\text{T-cell}}(Dose_{p}^{\text{Pt}})}$	$\frac{D(Dose_{p}^{*})}{D(Dose_{p}^{\text{Pt}})}$	$\frac{J_{2}(Dose_{p}^{*})}{J_{2}(Dose_{p}^{\text{Pt}})}$
1	0.94	1.11	1.18	1
2	0.95	0.82	1.62	1
3	0.97	1.05	1.09	1
4	0.98	1.1	1.25	1
5	0.98	1.07	1.2	1
6	0.94	0.89	1.2	1

In general, each of our optimal peptide vaccine doses was tailored accordingly to our level of preference. In the case of *J*_1_, we selected weight parameters to prioritize the overall tumor cell reduction (*A*_1_ = 1) over minimizing the vaccine dose (*B* = 1/3). Although we observe that for certain patients the overall optimal vaccine dose is almost double the clinical trial dose, the optimal vaccine dose does not pose any risk of toxicity since it is within the assumed safety range. The findings in Table 3 reflected this situation. On the other hand, for *J*_2_ our level of preference was the same among the overall tumor and immune responses as well as the total vaccine dose (*A*_1_ = 1, *A*_2_ = 1, *B* = 1), consistent with the observations in Table 4.

## Discussion

The optimization approach that we present here can help us identify an optimal peptide dose with the highest clinical benefit in overall tumor reduction and without excessive total vaccine dose when compared to another vaccine dose (e.g., the clinical trial dose). Our optimization approach starts by providing us with a set of optimal peptide doses to approximate a global minimizer for the objective functionals (*J*_1_ and *J*_2_). However, this global minimizer in Eq (9) may not provide the highest overall tumor reduction when compared to another vaccine dose, as illustrated in Fig 3. To overcome this challenge, we select an optimal vaccine dose from our set of optimal peptide doses with the highest tumor reduction when compared to the other vaccine dose. The selection process to identify this optimal dose is described by the two conditions in Eqs (13) and (14).

The vaccine dose optimization problem was applied to the set of six patients from an advanced melanoma cancer clinical trial [34]. We started by exploring the effect of changing a constant peptide dose on the initial and final tumor sizes to appropriately select the range for tested peptide doses in Fig 4. We selected a safe dose range (0.1x to 3x of the clinical trial dose) for the optimization, which reached a plateau at maximum tumor reduction for almost all patients. The weight parameters offer the context of the minimization. In the case of *J*_1_, the weights were chosen to prioritize tumor reduction (*A*_1_ = 1) over the vaccine dose (*B* = 1/3). In the case of *J*_2_, the focus was to have an equal weight on reduction in tumor size, number of activated T cells, and vaccine dose (*A*_1_ = 1, *A*_2_ = 1 and *B* = 1). We used the clinical trial dose of each patient to identify the optimal peptide dose with the highest clinical benefit in overall tumor reduction to illustrate our optimization approach.

The score of the optimal vaccine doses (for *J*_1_ and *J*_2_) were similar when compared to the score of the clinical trial dose (as shown in the last column of Tables 3 and 4). Despite this similarity in their scores, the optimal vaccine doses offered a higher clinical benefit in overall tumor reduction for almost every patient (as shown in the first column of Tables 3 and 4). Specifically, the total optimal vaccine dose for each patient was significantly lower when the objective functional *J*_2_ was used instead of *J*_1_. However, the overall tumor reduction was significantly higher (more clinical benefit) when the *J*_1_ optimal vaccine dose was used rather than *J*_2_. The additional minimization of the total number of activated T cells in *J*_2_ illustrated that the optimally predicted vaccine dose produced similar effects in reducing the total tumor size with less excessive immune response.

In contrast to the typical trend of decreasing doses during later vaccinations (booster phase) in Fig 5, an unexpected pattern emerged in the case of Patient 2. According to the *J*_1_-optimal analysis, it is optimal for Patient 2 to receive roughly 0.5 times the clinical trial dose for the first four and last vaccinations, while approximately 2.5 times the clinical trial dose is optimal for the vaccinations in between. This deviation from the expected pattern seen in other patients introduces an additional layer of complexity to our findings. The unique situation observed in Patient 2 suggests the possibility that the optimal starting time for administering the cancer vaccine might vary among patients. Additionally, when considering the *J*_2_-optimal approach, our findings indicate that Patients 2 and 6 with stage IV cancer, require higher doses for the initial prime vaccinations, followed by lower doses for subsequent administrations. This observation implies that when the goal is to minimize both the immune response and the tumor response, a “one size fits all” approach is not desirable and some patients benefit from higher initial vaccination dose that is subsequently decreased while others require low initial doses which are escalated.

We observe from *in silico* results in Tables 3 and 4 that with the help of an optimal vaccine (either from *J*_1_ or *J*_2_) in comparison to the clinical trial dose, there could have been higher overall tumor reductions among all patients. For Patients 2 and 6, cancer immunotherapy may reduce the final size of the tumor; however, the immunotherapy alone may not be sufficient to move these patients to a less advanced cancer stage as the model predicted a large tumor size at the end of the treatment period for all tested vaccine doses (including the optimal ones). In such patients, additional or alternative treatment options may be required to improve outcomes [8, 47].

In practice, it would be more realistic to minimize *J*_1_ than *J*_2_ since, to our knowledge, personalized cancer vaccines have not shown potential risks for safety or toxicity due to high T cell activation [34, 48, 49]. However, with *J*_2_, we explored the case when minimizing tumor and immune responses simultaneously can lead us to find vaccine doses that fit these two outcomes (with even lesser total vaccine dose). In this hypothetical scenario, the excessive T cell response from the cancer treatment could have negative consequences in the context of autoimmune diseases and tissue damage [39, 40]. Therefore, regulating and balancing T cell responses during treatment is essential for preventing and managing the adverse effects associated with excessive T cell activation.

## Limitations

Our study has several limitations. First, there are some limitations inherited from the MRM model [33]. The model did not consider the potential elimination of tumor cells by activated *CD*4^+^, the functions of memory and regulatory T cells, or tumor eradication by antigen-specific antibodies. Thus, these limitations are carried over to our work. Moreover, our optimization framework does not allow for optimization of the vaccination schedule. However, we observed that an optimal vaccine dose is usually a combination of higher and lower vaccine doses at some of the scheduled vaccination days. This could open a window of opportunity to explore different schedules where vaccination days requiring low doses (e.g., 0.1 of clinical trial dose) may be removed. Another limitation of our optimization problem is that it does not account for other combined treatments received by the patients. Patients 2 and 6 achieved a positive clinical outcome after receiving anti-programmed cell death protein 1 (anti-PD-1) antibody treatment post-vaccination [50]. In the future, it will be important to expand the model so that it accounts for combination therapy and longer outcome including pre/post immunotherapy treatment [48, 51, 52]. Furthermore, the model and optimization framework could be refined to incorporate other immunological mechanisms such as cancer relapse, resistance to immunotherapies, and immune escape that can offer a more comprehensive understanding of their dynamics. A significant limitation to applying our model in the clinical setting is that the optimization problem relies on the patient longitudinal data over the course of the treatment. Thus, it does not have a predictive value to help determine the vaccine dose prior to the treatment.

Determining the optimal personalized dose of a cancer vaccine is not a straightforward task. There are several logistical complexities involved in vaccine development that may render it impractical for manufacturers to implement the optimal dose. For instance, it may not be feasible to produce the exact optimal number of peptide molecules suggested by the model. The model’s fitting may be inaccurate due to insufficient data to estimate parameters and overfitting, resulting in unreliable and biased outcomes. However, our results offer a promising solution. One can target a cancer vaccine dose to be as effective as the optimal vaccine dose. In addition, our work has the potential to be integrated with a clinical trial, where the optimization framework presented here can be used to“learn” from the outcomes after the initial vaccine doses, and model parameters can be updated continuously over time to make predictions more accurate, like a feedback loop in the digital twin paradigm [53, 54].

## Conclusion

In this paper, we developed a dose optimization approach to find the optimal vaccine compositions, amount of peptides and adjuvant, that a patient’s vaccine requires to minimize two objective functionals (one intended for efficacy while another one for safety and efficacy) given a fixed peptide:adjuvant ratio and vaccination schedule. This study provides a potential pathway for investigating various dosing regimens in personalized immunotherapy and underscores the significance of comprehending the impacts of alternative doses in accomplishing the primary objectives of immunotherapy, namely, triggering a potent immune response to reduce or eliminate tumors.

The findings of our approach suggest that determining the optimal vaccine dose based on safety and efficacy could assist clinicians in developing and utilizing cancer vaccine therapies more effectively for each patient. The targeting of optimal vaccine doses, as outlined in this paper, could serve as a valuable tool for personalized cancer vaccine treatment in a clinical trial setting.

## Disclaimer

This article reflects the views of the authors and should not be construed to represent FDA’s views or policies.

## Supporting information

S1 Appendix Model description, necessary conditions and tables of numerical results. (ZIP)
