# Supplementary material for: Dose optimization of an adjuvanted peptide-based personalized neoantigen melanoma vaccine
Source: PLoS Comput Biol. 2024 Mar 1;20(3):e1011247. doi: 10.1371/journal.pcbi.1011247 (PMC10936818; doi:10.1371/journal.pcbi.1011247)
Supplement: S1 Appendix — (ZIP) [file pcbi.1011247.s001.zip › S1_Appendix.pdf]

Dose Optimization of a Personalized Neoantigen Peptide Cancer Vaccine for Melanoma Patients

Wencel Valega-Mackenzie[1], Marisabel Rodriguez-Messan[1], Osman N. Yogurtcu[1], Ujwani Nukala[1], Zuben E. Sauna[2], and Yang Hong[1,*]

[1]Office of Biostatistics and Pharmacovigilance, Center for Biologics Evaluation and Research, U.S. Food and Drug Administration, Silver Spring, Maryland, United States of America

[2]Office of Tissues and Advanced Therapies, Center for Biologics Evaluation and Research, U.S. Food and Drug Administration, Silver Spring, Maryland, United States of America

[*]Corresponding author: Hong.Yang@fda.hhs.gov

S1 Appendix. Model description, parameter values, necessary conditions and tables of numerical results.

Model description

Vaccine: Peptide and Adjuvant

The personalized cancer vaccine is formed by combining a mixture of peptide and adjuvant concentrations. Equations (1) and (2) measure the rate of change of peptides and adjuvant from dose administration to absorption by dendritic cells. The adjuvant works as an immunostimulatory agent that activates the innate immune system and allows maturation of dendritic cells. Peptides are then presented at the endosome of mature dendritic cells to interact with Major Histocompatibility Complex (MHC) class I and II molecules.

$$\frac{dp}{dt} = \underbrace{h_p(t)}_{\text{Peptide Dose Administration}} - \underbrace{\alpha_p p}_{\text{Absorption by DCs}} \tag{1}$$

$$\frac{dA_d}{dt} = \underbrace{h_a(t)}_{\text{Adjuvant Dose Administration}} - \underbrace{\alpha_d A_d}_{\text{Absorption by DCs}} \tag{2}$$

where

$$h_x(t) = \sum_{i=1}^{\tau} Dose_x \cdot \delta(t - \tau_i) \text{ for } x = a, p$$

represents the same vaccine dose ($Dose_x$ for $x = p, a$ is constant) a patient receives at the days of the vaccination schedule, τ_i for $i = 1, 2, 3, ..., \tau$, with τ as the total number of vaccination days and δ is the Dirac delta function.

Antigen presenting cells by dendritic cells (DCs)

Equations (3-4) model the dynamics of immature and mature DCs at the cellular level. In absence of a neoantigen cancer vaccine the immature DCs, D_I, have a logistic growth at the subcutaneous connective tissue with maximum intrinsic growth rate, Λ, and a carrying capacity of K_{DC}. In presence of the adjuvant, immature DCs differentiate into mature DCs, D_M. The parameters r_D and K_a represent the highest differentiation rate and half-saturation effect produced by the interaction with the adjuvant. Mature DCs can die naturally at a rate of δ_M.

$$\frac{dD_I}{dt} = \underbrace{\Lambda D_I\Big(1 - \frac{D_I}{K_{DC}}\Big)}_{\text{Logistic growth}} - \underbrace{\frac{r_D A_d}{K_a + A_d} D_I}_{\text{Maturation by adjuvant}} \tag{3}$$

$$\frac{dD_M}{dt} = \frac{r_D A_d}{K_a + A_d} D_I - \underbrace{\delta_M D_M}_{\text{Natural death}} \tag{4}$$

Processing and presentation of neoantigen by mature DCs

The rate of change of peptides in the endosome of mature DCs is described by Equation (5).

$$\frac{dp^E}{dt} = \alpha_p^E p \frac{V_E}{V_{sc}} \underbrace{- p^E \cdot \sum_s k_{\text{on},s} \cdot \frac{M_s^E}{V^E}}_{\text{Binding to MHC-I/II}} + \underbrace{\sum_s k_{\text{off},s} \cdot pM_s^E}_{\text{Dissociation of } p\text{-MHC-I/II}} - \underbrace{\beta_p p^E}_{\text{Degradation}}, \quad s = j,k \tag{5}$$

Initially, vaccine peptides are endocytosed at a rate $\alpha_p^E \frac{V_E}{V_{sc}}$ where the fraction $\frac{V_E}{V_{sc}}$ corresponds to the volume of endosomes in a single DC. After peptide endocytosis, endosomal peptides, p^E, can bind to endosomal MHC-I or MHC-II, in this case the new molecules are pM_s^E. Subsequently, the p-MHC molecules can dissociate into its original components at a rate $k_{\text{off},s}$. The effective dissociation constant

$$K_{D,s}^{\text{eff}} = \Big(\sum_{m=1}^{N} \frac{1}{K_{Dm,s}}\Big)^{-1}, \; s = j,k$$

is used to measure the interaction between the peptides and MHC-I allele type j or MHC-II allele type k. The constant $K_{D_m,s}$ corresponds to the binding affinities for $m = 1, 2, ..., N$ where N is the total number of immunogenic peptides. Moreover, the effective dissociation constant provide a relationship between the off/on bio-molecular rates, $K_{D,s} = \frac{k_{\text{off},s}}{k_{\text{on},s}}$[15].

Next in Equation (6) we describe the dynamics of endosomal peptide free MHC type I/II molecules.

$$\frac{dM_s^E}{dt} = \underbrace{\beta_M(M_s^E(0) - M_s^E)}_{\text{Homeostatic growth}} \underbrace{- k_{\text{on},s} \cdot p^E \frac{M_s^E}{V^E} + k_{\text{off},s} \cdot pM_s^E}_{\text{Binding between } p^E\text{'s and } M_s^E\text{'s}} + \underbrace{k_{in} \cdot M_s}_{\text{Recycling}}, \quad s = j,k \tag{6}$$

First, homeostatic growth of free MHC molecules is considered with intrinsic growth rate β_M and initial total number of MHC free molecules $M_s^E(0)$. Endosomal MHC I/II molecules can bind to endosomal peptides at a rate $k_{\text{on},s}$ or unbind at a rate $k_{\text{off},s}$. Lastly, MHC I/II from the cell membrane can get recycled into DC's endosome at a rate k_{in}.

Equation (7) models the dynamics of endosomal peptides bounded to MHC I/II for allele $s = j, k$, pM_s^E.

$$\frac{dpM_s^E}{dt} = \underbrace{k_{\text{on},s} \cdot p^E \frac{M_s^E}{V^E} - k_{\text{off},s} \cdot pM_s^E}_{\text{Formation and dissociation of } pM_s^E} - \underbrace{\beta_{pM} \cdot pM_s^E}_{\text{Degradation}} - \underbrace{k_{ext} pM_s^E}_{\text{Exocytosis}}, \quad s = j, k \tag{7}$$

These bounded molecules can dissociate into their original components, suffer from degradation at a rate β_{pM} or exocytosis onto the cell membrane at a rate k_{ext}. Exocytosed molecules are later recognized by T cells.

Equations (8) and (9) describe the rate of changes of peptide bounded MHC I/II, pM_s, and peptide free MHC I/II, M_s on the membrane of DC. These p-MHC molecules can dissociate into MHC molecules. Additionally, MHC molecules can also recycle back to the endosome.

$$\frac{dpM_s}{dt} = \underbrace{k_{ext} pM_s^E}_{\text{Formation of } pM_s} - \underbrace{k_{\text{off},s} \cdot pM_s}_{\text{Dissociation}}, \quad s = j, k \tag{8}$$

$$\frac{dM_s}{dt} = \underbrace{k_{\text{off},s} \cdot pM_s}_{\text{Formation of } M_s \text{ from dissociated } pM_s} - \underbrace{k_{in} \cdot M_s}_{\text{Recycling}}, \quad s = j, k \tag{9}$$

The total number of peptide bounded MHC I/II is determined by

$$pM_n(t) = N_A \cdot \sum_s pM_s(t) \cdot 10^{-12} \text{ for } (s, n) = \{(j, \text{I}), (k, \text{II})\}$$

where N_A is the Avogadro's number. The above formula provides the number of total p-MHC molecules, either type I or II, that are recognizable by naive and activated $CD4^+$ and $CD8^+$ T cells.

Tumor Cells

The dynamics of the tumor cells, T, is shown in Equation (10). In absence of immunotherapy, the tumor is assumed to have a logistic growth with an intrinsic tumor growth rate of r and a carrying capacity of (K_T is the highest possible number of tumor cells). However, in presence of a cancer vaccine, it is possible to eliminate tumor cells by the activation of $CD8^+$ T cells at a rate $\mathcal{D}$. This killing rate ($\mathcal{D}$) represents the interaction between the tumor cells and the immune response.

$$\frac{dT}{dt} = \underbrace{rT\Big(1 - \frac{T}{K_T}\Big)}_{\text{Logistic growth}} - \underbrace{\mathcal{D}T}_{\text{Elimination by } A_{CD8} \text{ T cells}} \tag{10}$$

This tumor killing rate $\mathcal{D}$ is known as dePillis-Radunskaya Law [1, 2, 5] and scientists have been using in it the literature [5, 10] to model or estimate the interaction between the tumor and the immune response either by natural killer cells or $CD8^+$ T cells. The parameters d, λ and s in the equation below measure the lysis rate, the A_{CD8}/tumor ratio rate and the half-maximum killing rate, respectively.

$$\mathcal{D} = d \frac{(\frac{A_{CD8}}{T})^\lambda}{s + (\frac{A_{CD8}}{T})^\lambda}$$

Helper and cytotoxic T cells

The dynamics of naive $CD4^+$ and $CD8^+$ T cells are described by Equation (11). Both naive T cell populations, N_{CDi}, have a logistic growth with intrinsic growth rate and carrying capacity b_i and K_{TCi} for $i = 4, 8$. In addition, they could also die naturally at a rate μ.

$$\frac{dN_{CDi}}{dt} = \underbrace{b_i N_{CDi}\Big(1 - \frac{N_{CDi}}{K_{TCi}}\Big)}_{\text{Logistic growth}} - \underbrace{\sigma_i F_{Pi} N_{CDi} \Psi_i}_{\text{Activation via mature DCs}} - \underbrace{\mu N_{CDi}}_{\text{Natural death}}, \quad i = 4, 8 \tag{11}$$

Moreover, naive T cells can recognize peptide bounded MHC type I/II on the mature DC's membrane. This process is capture by the product of $F_{pi}N_{CDi}$, in which case F_{p_i} for $i = 4, 8$ represents the frequency of specific neoantigens associated with $CD4^+$ or $CD8^+$ T cells. The function Ψ_i for $i = 4, 8$ represents the activation of neoantigen specific T cells by the interaction of mature DCs to naive and activated T cells as well as the number of p-MHC molecules as shown in the equation below:

$$\Psi_i = \left(\frac{D_M}{D_M + F_{p_i} N_{CDi} + A_{CDi}}\right)\left(\frac{pM_n}{pM_n + K_{pM}}\right) \text{ for } (i, n) = \{(4, \text{II}), (8, \text{I})\}.$$

The constant K_pM represents the half saturation number of p-MHC molecules. Note that the activation function Ψ_i always takes values in the closed interval $[0, 1]$. When activation is successful, naive T cells become activated, that is, they progress to A_{CD4} or A_{CD8} compartments.

The dynamics of activated $CD4^+$ and $CD8^+$ T cells are shown in Equations (12) and (13). After successful activation of naive T cell by mature DCs, activated T cells, A_{CDi} for $i = 4, 8$, are expected to grow. The A_{CD4} population can also grow via interaction with the tumor and it is model with the Michaelis-Menten interaction term $c_4 \frac{T}{a_1+T}$. Here, the constant c_4 corresponds to the antigenicity of the tumor while a_1 is the half saturation number of tumor cells.

$$\begin{aligned}\frac{dA_{CD4}}{dt} = {} & \underbrace{\sigma_4 F_{P4} N_{CD4} \Psi_4}_{\text{Activation via mature DCs}} + \underbrace{\rho_4 \Phi_4 A_{CD4}}_{\text{Proliferation/differentiation of } A_{CD4}} \\ & + \underbrace{c_4 \frac{T}{a_1 + T} A_{CD4}}_{\text{Proliferation due to tumor cells}} - \underbrace{\mu_4 A_{CD4}}_{\text{Natural death}}\end{aligned} \tag{12}$$

Similarly, the A_{CD8} population can increase its size by directly interacting with the tumor cells. This dynamics is captured by the first term in Equation (13). Here, we also use the Michaelis-Menten interaction term, but instead of T as in the A_{CD4} equation, with $\mathcal{D}^2T^2$. Further, it is also assumed that the A_{CD8} population can grow by the mass action interaction between activated $CD4^+$ and tumor cells. Both activated T cell populations can die at a rate μ_i for $i = 4, 8$.

$$\begin{aligned}\frac{dA_{CD8}}{dt} = {} & \underbrace{c\frac{\mathcal{D}^2T^2}{a + \mathcal{D}^2T^2} A_{CD8}}_{\text{Recruitment of } A_{CD8} \text{ due to tumor}} + \underbrace{c_8 A_{CD4} T}_{\text{Activation via interaction of } A_{CD4} \text{ and tumor cells}} \\ & + \underbrace{\sigma_8 F_{P8} N_{CD8} \Psi_8}_{\text{Activation via mature DCs}} + \underbrace{\rho_8 \Phi_8 A_{CD8}}_{\text{Proliferation/differentiation of } A_{CD8}} - \underbrace{\mu_8 A_{CD8}}_{\text{Natural death}}\end{aligned} \tag{13}$$

Lastly, both activated T cell populations can proliferate or differentiate depending on the sign of the function

$$\Phi_i = \left(\frac{D_M}{D_M + F_{p_i} N_{CDi} + A_{CDi}}\right)\left(\frac{pM_n - K_{pM}}{pM_n + K_{pM}}\right) \text{ for } (i, n) = \{(4, \text{II}), (8, \text{I})\}.$$

Proliferation of activated T cells occur when the total number of recognizable p-MHC I/II is bigger than K_{pM}. However, when pM_n is smaller than K_{pM} for n = I,II, differentiation of activated T cells occur.

Note that the rate of peptide and adjuvant presented above will not be part of the state system. Instead, we use the rate of peptide and adjuvant presented in the main manuscript along with Equations (3)-(13).

Necessary conditions and optimal control characterization

In order to derive the necessary conditions that an optimal concentration of peptide, $Dose_p^*(t)$, must satisfy for each minimization problem, we use Pontryagin's Maximum Principle [11]. By Pontryagin's Maximum Principle, the minimization problem using only objective functional J_2 is equivalent to minimizing the following Hamiltonian over the control variable $Dose_p(t)$,

$$\begin{aligned} H(t, x, Dose_p, \lambda) = A_1 \cdot T + A_2 \cdot A_{CD4} + A_3 \cdot A_{CD8} + B \cdot Dose_p^2 + \Big(\lambda_p p' + \lambda_{A_d} A_d' \\ + \lambda_{D_I} D_I' + \lambda_{D_M} D_M' + \lambda_{p^E} p^{E'} + \lambda_{M_s^E} M_s^{E'} + \lambda_{pM_s^E} pM_s^{E'} + \lambda_{pM_s} pM_s' \\ + \lambda_{M_s} M_s' + \lambda_T T' + \lambda_{N_{CD4}} N_{CD4}' + \lambda_{N_{CD8}} N_{CD8}' + \lambda_{A_{CD4}} A_{CD4}' \\ + \lambda_{A_{CD8}} A_{CD8}'\Big) \end{aligned} \tag{14}$$

where $x = (p, A_d, D_I, D_M, p^E, M_s^E, pM_s^E, pM_s, M_s, T, N_{CD4}, N_{CD8}, A_{CD4}, A_{CD8})$ is the vector of states, $\lambda = (\lambda_p, \lambda_{A_d}, \lambda_{D_I}, \lambda_{D_M}, \lambda_{p^E}, \lambda_{M_s^E}, \lambda_{pM_s^E}, \lambda_{pM_s}, \lambda_{M_s}, \lambda_T, \lambda_{N_{CD4}}, \lambda_{N_{CD8}}, \lambda_{A_{CD4}}, \lambda_{A_{CD8}})$ is the adjoint vector of each associated state, for $s = j, k$, and $Dose_p$ is any acceptable control. Observe that the prime notation ($'$) used to formulate the Hamiltonian describes the rate of change over time ($\frac{d}{dt}$). Our Hamiltonian in (14) can be expressed in a most general form as

$$H(t, x, Dose_p, \lambda) = \text{Integrand of } J_2 + \text{Adjoint} \times \text{RHS of State System} .$$

To find the adjoint system of differential equations, we take the derivative of the Hamiltonian with respect to each state such that

$$-\lambda'_{\text{state}} = -\frac{d(\lambda_{\text{state}})}{dt} = \frac{\partial H}{\partial \text{state}} \quad \text{for all states.}$$

The following system of equations corresponds to the adjoint system associated to the state system:

Adjoint equations for vaccine peptide and adjuvant concentrations

$$\begin{aligned} -\frac{d\lambda_p}{dt} &= -\alpha_p \lambda_p + \alpha_p^E \frac{V_E}{V_{sc}} \lambda_{p^E} \\ -\frac{d\lambda_{A_d}}{dt} &= -\alpha_d \lambda_{A_d} - \left(\frac{r_D K_a D_I}{(K_a + A_d)^2}\right)(\lambda_{D_I} - \lambda_{D_M}) \end{aligned} \tag{15}$$

Adjoint equations for antigen presenting cells to dendritic cells

$$\begin{aligned} -\frac{d\lambda_{D_I}}{dt} &= \Lambda\Big(1 - \frac{2D_I}{K_{DC}}\Big)\lambda_{D_I} - \frac{r_D A_d}{K_a + A_d}(\lambda_{D_I} - \lambda_{D_M}) \\ -\frac{d\lambda_{D_M}}{dt} &= -\delta_M \lambda_{D_M} - \sum_{i=4,8} \sigma_i F_{P_i} N_{CDi} \frac{\partial \Psi_i}{\partial D_M}(\lambda_{N_{CDi}} - \lambda_{A_{CDi}}) \\ &+ \sum_{i=4,8} \rho_i \frac{\partial \Phi_i}{\partial D_M} A_{CDi} \lambda_{A_{CDi}} \end{aligned} \tag{16}$$

where

$$\frac{\partial \Psi_i}{\partial D_M} = \left(\frac{F_{p_i}N_{CDi} + A_{CDi}}{(D_M + F_{p_i}N_{CDi} + A_{CDi})^2}\right)\left(\frac{pM_n}{pM_n + K_{pM}}\right)$$

$$\frac{\partial \Phi_i}{\partial D_M} = \left(\frac{F_{p_i}N_{CDi} + A_{CDi}}{(D_M + F_{p_i}N_{CDi} + A_{CDi})^2}\right)\left(\frac{pM_n - K_{pM}}{pM_n + K_{pM}}\right)$$

for $(i, n) \in \{(4, \mathrm{II}), (8, \mathrm{I})\}$. The partial derivatives above represent the rate of change produced by mature DCs on the activation and proliferation/differentiation functions of naive or activated T cells.

Adjoint equation for antigen processing and presentation cells by mature dendritic cells

$$\begin{aligned}
-\frac{d\lambda_{p^E}}{dt} &= -\left(\sum_s k_{\mathrm{on},s}\cdot\frac{M_s^E}{V^E}\right)\lambda_{p^E} - \left(\sum_s k_{\mathrm{on},s}\frac{M_s^E}{V^E}(\lambda_{M_s^E} - \lambda_{pM_s^E})\right) - \beta_p\lambda_{p^E} \\
-\frac{d\lambda_{M_s^E}}{dt} &= -p^E\frac{k_{\mathrm{on},s}}{V^E}(\lambda_{p^E} + \lambda_{M_s^E} - \lambda_{pM_s^E}) - \beta_M\lambda_{M_s^E} \\
-\frac{d\lambda_{pM_s^E}}{dt} &= k_{\mathrm{off},s}(\lambda_{p^E} + \lambda_{M_s^E} - \lambda_{pM_s^E}) - k_{ext}(\lambda_{pM_s^E} - \lambda_{pM_s}) - \beta_{pM}\lambda_{pM_s^E} \\
-\frac{d\lambda_{pM_s}}{dt} &= k_{\mathrm{off},s}(\lambda_{M_s} - \lambda_{pM_s}) - \sum_{i=4,8}\sigma_i F_{P_i}N_{CDi}\frac{\partial\Psi_i}{\partial pM_s}(\lambda_{N_{CDi}} - \lambda_{A_{CDi}}) \\
&\quad + \sum_{i=4,8}\rho_i\frac{\partial\Phi_i}{\partial pM_s}A_{CDi}\lambda_{A_{CDi}} \\
-\frac{d\lambda_{M_s}}{dt} &= k_{in}(\lambda_{M_s^E} - \lambda_{M_s})
\end{aligned} \tag{17}$$

for $s = j, k$ with j and k representing the number of alleles that bind to MHC molecules class I and II, respectively. Moreover,

$$\frac{\partial \Psi_i}{\partial pM_s} = \left(\frac{D_M}{D_M + F_{p_i}N_{CDi} + A_{CDi}}\right)\left(\frac{N_a K_{pM}10^{-12}}{(pM_n + K_{pM})^2}\right)$$

$$\frac{\partial \Phi_i}{\partial pM_s} = \left(\frac{D_M}{D_M + F_{p_i}N_{CDi} + A_{CDi}}\right)\left(\frac{2N_a K_{pM}10^{-12}}{(pM_n + K_{pM})^2}\right)$$

for $(i, n) \in \{(4, \mathrm{II}), (8, \mathrm{I})\}$. The partial derivatives above represent the rate of change produced by p-MHC type I or II allele s on the activation and proliferation/differentiation functions of naive or activated T cells.

Adjoint equation for tumor cells

$$\begin{aligned}
-\frac{d\lambda_T}{dt} &= A_1 + \left(r - \frac{2rT}{K_T} - \mathcal{D} - T\frac{\partial\mathcal{D}}{\partial T}\right)\lambda_T + \left(c_4\frac{a_1}{(a_1+T)^2}A_{CD4}\right)\lambda_{A_{CD4}} \\
&\quad + \left(c\frac{2a\mathcal{D}^2 T}{(a + \mathcal{D}^2T^2)^2}\frac{\partial\mathcal{D}}{\partial T}A_{CD8} + c_8 A_{CD4}\right)\lambda_{A_{CD8}}
\end{aligned} \tag{18}$$

where

$$\mathcal{D} = d\frac{(\frac{A_{CD8}}{T})^\lambda}{s + (\frac{A_{CD8}}{T})^\lambda}$$

is the tumor killer rate due to the interaction of the tumor cells and CD8+ T cells, and

$$\frac{\partial\mathcal{D}}{\partial T} = -d\frac{\frac{\lambda s A_{CD8}}{T^2}(\frac{A_{CD8}}{T})^{\lambda-1}}{(s+(\frac{A_{CD8}}{T})^{\lambda})^2}$$

describes the rate of change of the immune system killing rate with respect to the tumor cells.
Adjoint equations for helper and cytotoxic cells

$$\begin{aligned}
-\frac{d\lambda_{N_{CDi}}}{dt} &= \Big(b_i - 2b_i\frac{N_{CDi}}{K_{TCi}}\Big)\lambda_{N_{CDi}} - \Big(\sigma_i F_{Pi}\Psi_i + \sigma_i F_{Pi} N_{CDi}\frac{\partial\Psi_i}{\partial N_{CDi}}\Big)(\lambda_{N_{CDi}} - \lambda_{A_{CDi}})\\
&\quad + \rho_i\frac{\partial\Phi_i}{\partial N_{CDi}}A_{CDi}\lambda_{A_{CDi}} - \mu\lambda_{N_{CDi}}\\
-\frac{d\lambda_{A_{CD4}}}{dt} &= A_2 - \sigma_4 F_{P4} N_{CD4}\frac{\partial\Psi_4}{\partial A_{CD4}}(\lambda_{N_{CD4}} - \lambda_{A_{CD4}}) + \rho_4\Phi_4\lambda_{A_{CD4}}\\
&\quad + \Big(\rho_4 A_{CD4}\frac{\partial\Phi_4}{\partial A_{CD4}} + c_4\frac{T}{a_1+T} - \mu_4\Big)\lambda_{A_{CD4}} + c_8 T\lambda_{A_{CD8}} \qquad (19)\\
-\frac{d\lambda_{A_{CD8}}}{dt} &= A_3 - T\frac{\partial\mathcal{D}}{\partial A_{CD8}}\lambda_T - \sigma_8 F_{P8} N_{CD8}\frac{\partial\Psi_8}{\partial A_{CD8}}(\lambda_{N_{CD8}} - \lambda_{A_{CD8}})\\
&\quad + \Big(c\frac{\mathcal{D}^2T^2}{a+\mathcal{D}^2T^2} + cA_{CD8}\frac{2a\mathcal{D}T^2}{(a+\mathcal{D}^2T^2)^2}\frac{\partial\mathcal{D}}{\partial A_{CD8}}\Big)\lambda_{A_{CD8}}\\
&\quad + \Big(\rho_8\Phi_8 + \rho_8 A_{CD8}\frac{\partial\Phi_8}{\partial A_{CD8}} - \mu_8\Big)\lambda_{A_{CD8}}
\end{aligned}$$

where

$$\begin{aligned}
\frac{\partial\Psi_i}{\partial N_{CDi}} &= \left(\frac{-D_M F_{p_i}}{(D_M + F_{p_i}N_{CDi} + A_{CDi})^2}\right)\left(\frac{pM_n}{pM_n + K_{pM}}\right)\\
\frac{\partial\Phi_i}{\partial N_{CDi}} &= \left(\frac{-D_M F_{p_i}}{(D_M + F_{p_i}N_{CDi} + A_{CDi})^2}\right)\left(\frac{pM_n - K_{pM}}{pM_n + K_{pM}}\right)\\
\frac{\partial\Psi_i}{\partial A_{CDi}} &= \left(\frac{-D_M}{(D_M + F_{p_i}N_{CDi} + A_{CDi})^2}\right)\left(\frac{pM_n}{pM_n + K_{pM}}\right)\\
\frac{\partial\Phi_i}{\partial A_{CDi}} &= \left(\frac{-D_M}{(D_M + F_{p_i}N_{CDi} + A_{CDi})^2}\right)\left(\frac{pM_n - K_{pM}}{pM_n + K_{pM}}\right)\\
\frac{\partial\mathcal{D}}{\partial A_{CD8}} &= d\frac{\frac{\lambda s}{T}(\frac{A_{CD8}}{T})^{\lambda-1}}{(s+(\frac{A_{CD8}}{T})^{\lambda})^2}
\end{aligned}$$

are the partial derivatives corresponding to the rate of change produced by naive or activated T cells on the activation and proliferation/differentiation functions of naive or activated T cells.

The adjoint system consists of differential equations in (16), (17),(18) and (19) subject to final time conditions (transversality condition),

$$\lambda_{\text{state}}(t_f) = \begin{cases} 0 & \text{if state } \neq T\\ A_1 & \text{if state } = T\end{cases} \qquad (20)$$

where T is the state associated with the number of tumor cells. The reason behind the final time condition for λ_T is different than 0 comes from the payoff term ($T(t_f)$) in the objective

functional. More details on how this condition is obtained can be found in [7] for more general payoff terms.

To find the optimal vaccine dose characterization, we differentiate the Hamiltonian (14) with respect to each peptide and adjuvant dose variables, that is:

$$\frac{\partial H}{\partial Dose_p}\Big|_{Dose_p=Dose_p^*} = 2BDose_p^* + \rho\lambda_p + \rho\lambda_a \frac{\partial Dose_p^{mg}}{\partial Dose_p} = 0.$$

where $\frac{\partial Dose_p^{mg}}{\partial Dose_p} = \frac{MW}{10^6}$ and MW is the molecular weight of all peptides combined. The above equation is known as the *optimality condition.* After solving for $Dose_p^*$, and taking into account the lower and upper bounds, $Dose_p^l$ and $Dose_p^u$, for all acceptable peptide concentrations, $Dose_p(t)$, we can see that the optimal peptide concentrations (**optimal control characterization**) is given by

$$Dose_p^* = \min\left\{Dose_p^u, \max\left\{Dose_p^l, \frac{-\rho\Big(\lambda_p + \lambda_a \cdot MW \cdot 10^{-6}\Big)}{2B}\right\}\right\}. \tag{21}$$

The state and adjoint systems with corresponding initial and final time conditions, together with the optimal control characterization, is known as the *optimality system.*

Note that one can prove the following theorems associated with each optimal control problem.

Theorem 1. *There exists an optimal peptide dose $Dose_p^* \in \mathcal{V}$ that minimizes the objective functional J_1 (respectively for J_2) subject to the state system and non-negative initial conditions.*

Theorem 2. *Given an optimal concentration of peptide, $Dose_p^* \in \mathcal{V}$, with its corresponding states, then there is an adjoint system with transversality conditions. In addition, there is an optimal control characterization of the optimal pair in terms of state and adjoint functions.*

The first theorem can be proved using [3], showing the convexity of the integrand of each objective functional with respect to $Dose_p$, *a priori* estimates of the state solutions, and the Lipschitz property of the state system with respect to the state variables. Interested readers can see some of these references [14, 8] for examples, but we skip the proof of this theorem here due to its technicalities. However, we showed the existence of the adjoint system as well as the optimal control characterization to prove the second theorem.

Parameter values and definitions

In this section we present the parameters values used for the numerical simulations in each patient with their corresponding definitions.

	**Parameter**	**Definition**	**Value**	**Units**
**Vaccine**	ρ	Subcutaneous vaccination rate	1000	day^{-1}
	$r_{a:p}$	Adjuvant:Peptide ratio	patient-specific	-
	α_p	Internalization rate of peptides by DCs	0.28	day^{-1}
	α_d	Internalization rate of adjuvant by DCs	0.5	day^{-1}
	V_{sc}	Volume of injections site	$4 \times (0.1919)$	L

Table A – continued from previous page

	Parameter	Definition	Value	Units
**Dendritic cells**	Λ	Maximum growth rate	3.75	day^{-1}
	δ_M	Death rate of mature DCs	0.33	day^{-1}
	r_D	Maximum differentiation rate	2.48	day^{-1}
	K_a	Half-maximum adjuvant effect constant	6.64	-
	K_{DC}	Carrying capacity	2.38×10^7	cells
	V_E	Volume of endosomes in a DC	1×10^{-14}	L
**Antigen processing and presentation**	N_A	Avogadro's constant	6.02×10^{23}	mol^{-1}
	α_p^E	Endosomal internalization rate of peptides	70	day^{-1}
	β_p	Degradation rate of peptides	14.4	day^{-1}
	$k_{\text{on},1}$	On rate for T-epitope-MHCI binding with allele j	1.8144×10^{-2}	pM^{-1}·day^{-1}
	$k_{\text{off},j}$	Off rate for T-epitope-MHCI binding with allele j	$k_{\text{on},1} \cdot K_{D,j}^{\text{eff}}$	day^{-1}
	$k_{\text{on},2}$	On rate for T-epitope-MHCII binding with allele k	8.64×10^{-3}	pM^{-1}·day^{-1}
	$k_{\text{off},k}$	Off rate for T-epitope-MHCII binding with allele k	$k_{\text{on},2} \cdot K_{D,k}^{\text{eff}}$	day^{-1}
	β_M	Degradation rate of endosomal free MHCI/II molecules	1.663	day^{-1}
	k_{in}	Recycling rate of free MHC molecules	14.4	day^{-1}
	k_{ext}	Exocytosis rate of p-MHC complex	28.8	day^{-1}
	β_{pM}	Degradation rate of p-MHCI/II complex	0.166	day^{-1}
**T-cells**	a	Number of tumor cells needed for half-maximal A_{CD8} proliferation	5×10^7	cells
	a_1	Half saturation constant of the CD4$^+$ T-cells production rate	1×10^3	cells
	b_4	Max. growth rate of naïve CD4$^+$ T-cells	0.15	day^{-1}
	b_8	Max. growth rate of naïve CD8$^+$ T-cells	0.12	day^{-1}
	σ_4	Max. activation rate of naïve CD4$^+$ T-cells	1.5	day^{-1}
	σ_8	Max. activation rate of naïve CD8$^+$ T-cells	3	day^{-1}
	K_{TC}	Carrying capacity of T-cells	8.57×10^{11}	cells
	F_{P_4}	Frequency of antigen-specific CD4$^+$ T-cells	$\{0.006, 0.001, 0.001, 0.001, 0.003, 0.001\}$	-
	F_{P_8}	Freq. of antigen-specific CD8$^+$ T-cells	$\{0.007, 0.002, 0.006, 0.002, 0.002, 0.001\}$	-
	K_{pM}	Half-maximum effect of activation	400	-
	c	Maximum CD8$^+$ T-cells recruitment rate	patient-specific	day^{-1}
	c_4	Maximum CD4$^+$ T-cell production rate	patient-specific	day^{-1}

Table A – continued from previous page

	Parameter	Definition	Value	Units
	c_8	Rate at which CD8$^+$ T-cells are stimulated to be produced	6.5×10^{-11}	day^{-1}
	μ	Death rate of naïve T-cells	0.0029	day^{-1}
	μ_4	Death rate of activated CD4$^+$ T-cells	0.031	day^{-1}
	μ_8	Death rate of activated CD8$^+$ T-cells	0.022	day^{-1}
	ρ_4	Proliferation rate for activated T-cells	0.0265	day^{-1}
	ρ_8	Proliferation rate for activated CD8$^+$ T-cells	0.0509	day^{-1}
**Tumor cells**	r	Maximum growth rate	0.004	day^{-1}
	K_T	Carrying capacity of tumor	1.45×10^{10}	cells
	d	Max. lysis rate by activated T-cells	patient-specific	day^{-1}
	λ	Dependence of lysis rate on the effector/target ratio constant	patient-specific	-
	s	Half-maximal effect of tumor cell lysis	0.0839	-
**Initial Conditions**	$A_d(0)$	Adjuvant concentration in a vaccine	0	mg/L
	$p(0)$	Peptide amount in a vaccine	0	pmol
	$D_I(0)$	Immature dendritic cells	1×10^7	cells
	$D_M(0)$	Mature dendritic cells	0	cells
	$p^E(0)$	Endosomal peptides	0	pmol
	$M_j^E(0)$	Endosomal MHC-I	$(1.6, 5.8) \times 10^{-7}$	pmol
	$M_k^E(0)$	Endosomal MHC-II	$(0.16, 8.3) \times 10^{-8}$	pmol
	$pM_j^E(0)$	Endosomal p-MHCI complex	0	pmol
	$pM_k^E(0)$	Endosomal p-MHCII complex	0	pmol
	$pM_j(0)$	p-MHCI on DC membrane	0	pmol
	$pM_k(0)$	p-MHCII on DC membrane	0	pmol
	$M_j(0)$	Free MHC-I on DC membrane	0	pmol
	$M_k(0)$	Free MHC-II on DC membrane	0	pmol
	$N_{CD4}(0)$	Naïve CD4$^+$ T-cell count	$(5.38 \times 10^9)\times 0.7$	cells
	$N_{CD8}(0)$	Naïve CD8$^+$ T-cell count	$(5.38 \times 10^9) \times 0.3$	cells
	$A_{CD4}(0)$	Activated CD4$^+$ T-cell count	1.43×0.7	cells
	$A_{CD8}(0)$	Activated CD8$^+$ T-cell count	1.43×0.3	cells
	$T(0)$	Tumor cell count	patient-specific	cells

Table A: Parameter values and definitions including units. Taken and adapted from [13]. Note that pM or picomolar is pmol/L.

Table B: Patient specific parameters. Taken and adapted from [13].

Parameter	Patient 1	Patient 2	Patient 3	Patient 4	Patient 5	Patient 6
c	0.0156	0.0084	0.0448	0.0056	0.031	5.55×10^{-5}
c_4	0.04	0.0105	0.0487	0.0467	0.0327	0.042
d	0.068	0.0196	0.0649	0.0617	0.0525	0.035
λ	0.978	0.0105	0.0487	0.5	0.8568	0.6
$T(0)$	1.375×10^6	1.139×10^9	1.177×10^6	1.493×10^6	855265	1.078×10^9

Weight parameters

The constants A_1, A_2 and B are weight parameters that measure the relative importance of each term in the objective functionals. However, effectively balancing these non-negative weights to make the contributions of each term distinguishable is not a straightforward task. For a random choice of weights, it is likely that one of the terms dominates over the others. The choice of these weights may vary among patients due to individual differences in initial tumor sizes and immune responses to the vaccine [6]. See our sensitivity analysis section of the objective functionals on the weight parameters for our case study.

Generally, the higher the value of the weight, the higher the relative importance associated to that term. To avoid ambiguity on our weight choices, we normalize each weight by the corresponding contribution of J_T, $J_{\text{T-cells}}$ and J_V at the highest acceptable dose, $(Dose_p^u)$

$$A_1 = \frac{\hat{A}_1}{J_T(Dose_p^u)}, \quad A_2 = \frac{\hat{A}_2}{J_{\text{T-cells}}(Dose_p^u)}, \quad B = \frac{\hat{B}}{J_V(Dose_p^u)}.$$

We based our choice of the highest acceptable dose on the results from Fig 4 where patients achieved the smallest tumor size by the final time. In practice, one can estimate the normalizing values of J_T, $J_{\text{T-cells}}$ and J_V using *in vivo* data of the immune response to different vaccine doses or using early data on the treatment response after the first prime vaccinations.

Without loss of generality we drop the hats and assume that A_1, A_2 and B are in $[0, 1]$. The closer these weights are to 0 or 1 shows the low or high level of importance associated with that term. The normalizing values used for each patient in our case study are displayed in Table C.

Table C: Normalizing values use for the weights A_1, A_2 and B for J_T, $J_{\text{T-cell}}$ and J_V at 3-fold the clinical trial dose, $Dose_p^{\text{pt}}$.

Patient ID	$J_T(3 \cdot Dose_p^{\text{pt}})$	$J_{\text{T-cell}}(3 \cdot Dose_p^{\text{pt}})$	$J_V(3 \cdot Dose_p^{\text{pt}})$
Patient 1	3.41e08	6.65e12	1.14e09
Patient 2	8.70e12	4.46e11	1.12e09
Patient 3	3.09e08	5.46e12	1.35e09
Patient 4	2.79e08	7.05e12	1.19e09
Patient 5	3.86e08	6.89e12	1.32e09
Patient 6	1.69e11	1.37e11	1.29e09

Identifiability and sensitivity analyses

In this section we present a identifiability analysis on a constant peptide dose, $Dose_p$, and a sensitivity analysis of the objective functionals on their weight parameters.

Identifiability analysis

Identifiability analysis is a crucial tool in mathematical modeling for assessing whether model parameters can be uniquely determined from the data [4, 12]. In this study, we also conduct a structural and practical identifiability analysis for all patients with respect to the parameter $Dose_p$. First, we show that the parameter $Dose_p$ is structurally identifiable when fitted to error-free data by determining the true $Dose_p$ value through minimizing the squared error between the estimated and true solutions for the activated $CD4^+$ and $CD8^+$ T cells. Subsequently, leveraging the true model solutions for the activated $CD4^+$ and $CD8^+$ T cells, we generate synthetic noise data to facilitate the estimation of $Dose_p$. The objective now shifts to minimize the squared error between the estimated solution and the generated noisy data, thereby deducing the $Dose_p$ value that optimally aligns with the synthetic data. We classify the parameter $Dose_p$ as practically identifiable if the ratio between the estimated and true peptide concentrations closely approaches unity based on the added noise. The synthetic data are constructed by adding normally distributed errors with a mean of zero and a standard deviation equivalent to 10% of the standard deviation of the true solutions. Our numerical findings, presented in Fig A, demonstrate that the parameter $Dose_p$ exhibits practical identifiability across all patients, with matches of 97% or higher with the true $Dose_p$ values.

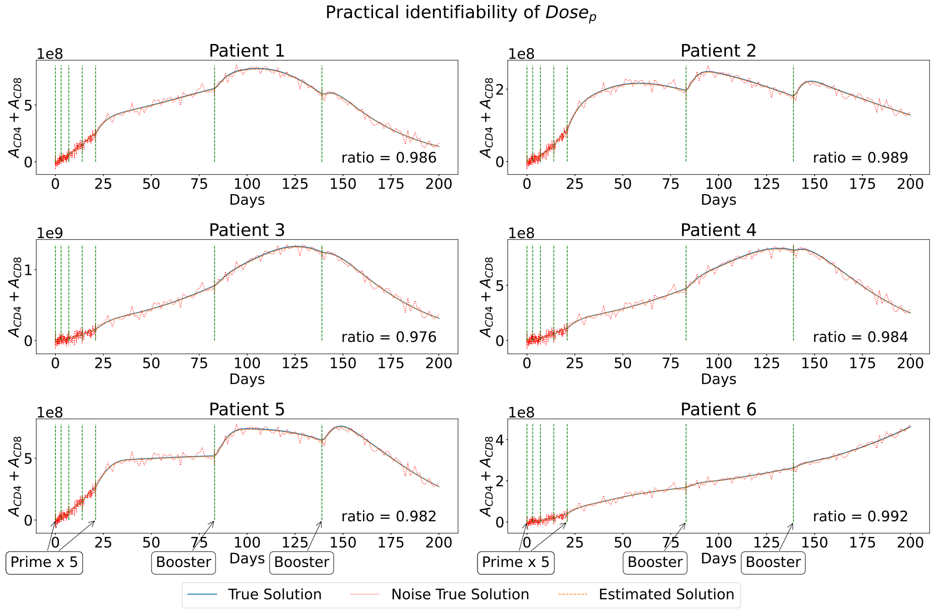

Figure A: Practical identifiability of constant $Dose_p$ using synthetic data of activated T cells. The ratio value corresponds to the ratio between estimated and true $Dose_p$ values.

Sensitivity analysis

A global sensitivity analysis is performed using Latin Hypercube Sampling (LHS) along with Partial Rank Correlation Coefficient (PRCC) to assess the sensitivity of objective functionals, J_1 and J_2, in response to variations in the constant values of $Dose_p$ and the weight parameters A_1, A_2, A_3 and B

when these are **not** normalized. The PRCC values, ranging from -1 and 1, indicate the correlation between input and output, with the magnitude representing the uncertainty in estimating the input's impact on outcome [9, 4]. The range of values for $Dose_p$ spans from 0.01 to 3 folds the clinical trial dose, and for weight parameters, the range is assumed from 10^{-5} to 10^4.

The PRCC values for all patients are depicted in Fig B. The analysis reveals high sensitivity of J_1 and J_2 to $Dose_p$ and B (weight associated to the dose) for Patients 1, 3, 4 and 5 (patients with stage III). Only patients with stage IV show negative correlation of J_1 to $Dose_p$. The analysis also shows that patients with stage III are not sensitive to A_1 (weight associated to tumor), whereas those with stage IV are sensitive. Moreover, only patients with stage III exhibit J_2 sensitivity to A_2 (weight associated to A_{CD4} and A_{CD8}) across all patients. We would like to remind the reader that the parameter A_2 is not in J_1.

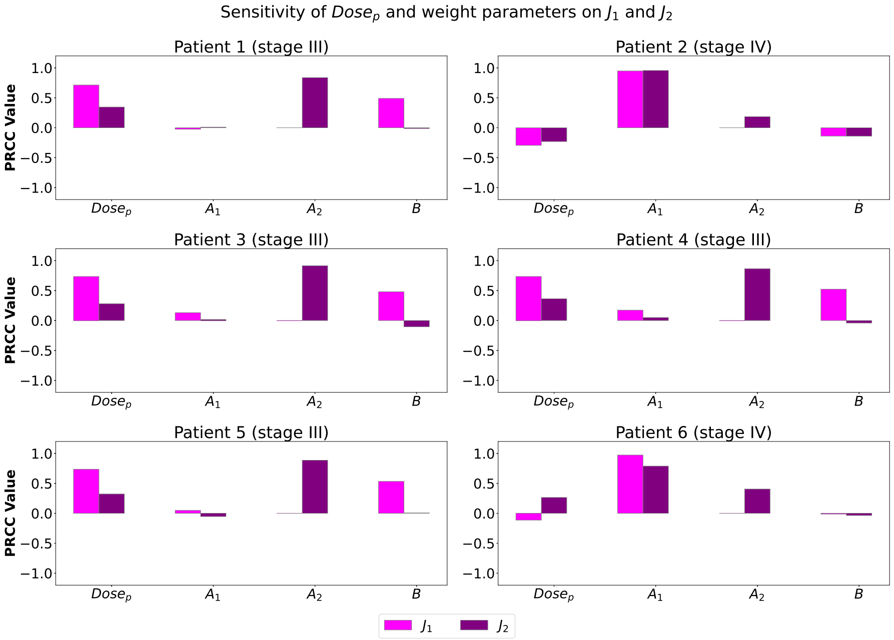

Figure B: Sensitivity of objective functionals J_1 and J_2. PRCC values for the constant peptide concentration, $Dose_p$ and not normalized weight parameters. The sensitivity of A_1 should be interpreted with caution as A_1 and the objective functional cost (either J_1 or J_2 score) have a strong positive correlation. Different increasing choices of A_1 would likely lead to a higher cost in J_1 or J_2 scores as the term, J_T, is likely to be dominant. In other words, the sensitivity of A_1 will skew any possible contributions from the other weights and the peptide dose to the score if not calibrated properly with the other weights. Note that the parameter A_2 is not in J_1.

In an effort to control these variability among the weight parameters, we normalized the weights for each patient using the values in Table C. With the normalized weights, we observe from Fig C that the new sensitivities of J_1 and J_2 to the vaccine dose and weights are similar among all the patients.

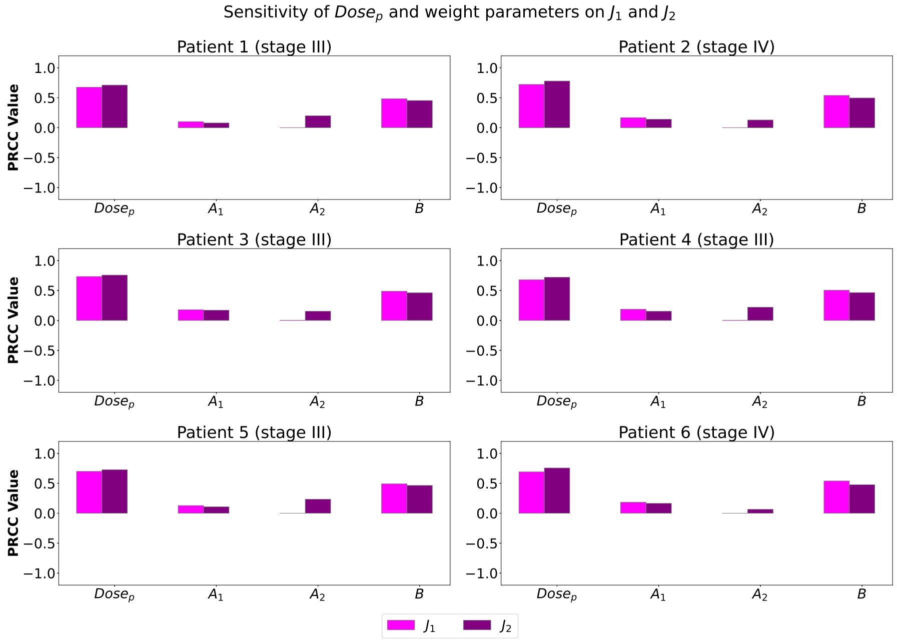

Figure C: Sensitivity of objective functionals J_1 and J_2 with normalized weights. PRCC values for the constant peptide concentration, $Dose_p$ and weight parameters. Note that the parameters A_2 is not in J_1.

Optimal vaccine doses using J_1

The baseline vaccine for each patient can be found in the table below followed by the optimal vaccine doses. Note that all the values in the optimal vaccine tables have been round to two decimal places.

Table D: Baseline vaccine dose for all patients

Patient ID	Peptide (mg/4ml)	Adjuvant(mg/4ml)
1	3.9	2
2	5.1	2
3	4.2	2
4	4.2	2
5	6	2
6	6	2

Table E: Optimal vaccine dose components for Patient 1 per vaccination day.

Patient 1	Day 0	Day 3	Day 7	Day 14	Day 21	Day 83	Day 139
Peptide (mg/4ml)	11.580	11.460	11.460	11.460	11.460	0.660	0.380
Adjuvant (mg/4ml)	5.940	5.880	5.880	5.880	5.880	0.340	0.200

Table F: Optimal vaccine dose components for Patient 2 per vaccination day.

Patient 2	Day 0	Day 3	Day 7	Day 14	Day 21	Day 83	Day 139
Peptide (mg/4ml)	0.510	0.500	0.500	0.500	12.490	11.300	2.100
Adjuvant (mg/4ml)	0.200	0.200	0.200	0.200	4.900	4.430	0.820

Table G: Optimal vaccine dose components for Patient 3 per vaccination day.

Patient 3	Day 0	Day 3	Day 7	Day 14	Day 21	Day 83	Day 139
Peptide (mg/4ml)	12.470	12.340	12.340	12.340	12.340	4.590	0.410
Adjuvant (mg/4ml)	5.940	5.880	5.880	5.880	5.880	2.190	0.200

Table H: Optimal vaccine dose components for Patient 4 per vaccination day.

Patient 4	Day 0	Day 3	Day 7	Day 14	Day 21	Day 83	Day 139
Peptide (mg/4ml)	9.350	9.260	9.260	9.260	9.260	9.260	1.620
Adjuvant (mg/4ml)	4.450	4.410	4.410	4.410	4.410	4.410	0.770

Table I: Optimal vaccine dose components for Patient 5 per vaccination day.

Patient 5	Day 0	Day 3	Day 7	Day 14	Day 21	Day 83	Day 139
Peptide (mg/4ml)	13.360	13.220	13.220	13.220	13.220	13.220	3.600
Adjuvant (mg/4ml)	4.450	4.410	4.410	4.410	4.410	4.410	1.200

Table J: Optimal vaccine dose components for Patient 6 per vaccination day.

Patient 6	Day 0	Day 3	Day 7	Day 14	Day 21	Day 83	Day 139
Peptide (mg/4ml)	17.810	17.630	17.630	17.630	12.060	0.590	0.590
Adjuvant (mg/4ml)	5.940	5.880	5.880	5.880	4.020	0.200	0.200

Optimal vaccine doses using J_2

Table K: Optimal vaccine dose components for Patient 1 per vaccination day.

Patient 1	Day 0	Day 3	Day 7	Day 14	Day 21	Day 83	Day 139
Peptide (mg/4ml)	5.790	5.730	5.730	5.730	5.730	2.570	0.380
Adjuvant (mg/4ml)	2.970	2.940	2.940	2.940	2.940	1.320	0.200

Table L: Optimal vaccine dose components for Patient 2 per vaccination day.

Patient 2	Day 0	Day 3	Day 7	Day 14	Day 21	Day 83	Day 139
Peptide (mg/4ml)	13.880	13.740	13.740	13.740	0.500	0.500	0.500
Adjuvant (mg/4ml)	5.440	5.390	5.390	5.390	0.200	0.200	0.200

Table M: Optimal vaccine dose components for Patient 3 per vaccination day.

Patient 3	Day 0	Day 3	Day 7	Day 14	Day 21	Day 83	Day 139
Peptide (mg/4ml)	5.190	5.140	5.140	5.140	5.140	5.140	0.410
Adjuvant (mg/4ml)	2.470	2.450	2.450	2.450	2.450	2.450	0.200

Table N: Optimal vaccine dose components for Patient 4 per vaccination day.

Patient 4	Day 0	Day 3	Day 7	Day 14	Day 21	Day 83	Day 139
Peptide (mg/4ml)	5.200	5.140	5.140	5.140	5.140	5.140	5.140
Adjuvant (mg/4ml)	2.470	2.450	2.450	2.450	2.450	2.450	2.450

Table O: Optimal vaccine dose components for Patient 5 per vaccination day.

Patient 5	Day 0	Day 3	Day 7	Day 14	Day 21	Day 83	Day 139
Peptide (mg/4ml)	7.420	7.340	7.340	7.340	7.340	7.340	5.350
Adjuvant (mg/4ml)	2.470	2.450	2.450	2.450	2.450	2.450	1.780

Table P: Optimal vaccine dose components for Patient 6 per vaccination day.

Patient 6	Day 0	Day 3	Day 7	Day 14	Day 21	Day 83	Day 139
Peptide (mg/4ml)	13.360	13.230	13.230	7.980	0.590	0.590	0.590
Adjuvant (mg/4ml)	4.450	4.410	4.410	2.660	0.200	0.200	0.200

References

[1] LG Depillis, A Eladdadi, and AE Radunskaya. "Modeling cancer-immune responses to therapy". In: *Journal of pharmacokinetics and pharmacodynamics* 41.5 (2014), pp. 461–478.

[2] Lisette DePillis, Angela Gallegos, and Ami Radunskaya. "A model of dendritic cell therapy for melanoma". In: *Frontiers in oncology* 3 (2013), p. 56.

[3] Wendell H Fleming and Raymond W Rishel. *Deterministic and stochastic optimal control.* Vol. 1. Springer Science & Business Media, 2012.

[4] Jill Gallaher et al. "Methods for determining key components in a mathematical model for tumor–immune dynamics in multiple myeloma". In: *Journal of Theoretical Biology* 458 (2018), pp. 31–46.

[5] Lifeng Han et al. "Analysis of tumor-immune functional responses in a mathematical model of neoantigen cancer vaccines". In: *Mathematical Biosciences* (2023), p. 108966.

[6] Angela M Jarrett et al. "Optimal control theory for personalized therapeutic regimens in oncology: Background, history, challenges, and opportunities". In: *Journal of clinical medicine* 9.5 (2020), p. 1314.

[7] Suzanne Lenhart and John T Workman. *Optimal control applied to biological models.* CRC Press, 2007.

[8] Abhishek Mallela, Suzanne Lenhart, and Naveen K Vaidya. "HIV–TB co-infection treatment: Modeling and optimal control theory perspectives". In: *Journal of Computational and Applied Mathematics* 307 (2016), pp. 143–161.

[9] Simeone Marino et al. "A methodology for performing global uncertainty and sensitivity analysis in systems biology". In: *Journal of theoretical biology* 254.1 (2008), pp. 178–196.

[10] Oleg Milberg et al. "A QSP model for predicting clinical responses to monotherapy, combination and sequential therapy following CTLA-4, PD-1, and PD-L1 checkpoint blockade". In: *Scientific reports* 9.1 (2019), pp. 1–17.

[11] LS Pontryagin et al. *Mathematical Theory of Optimal Processes.* Wiley, New York, 1962.

[12] Andreas Raue et al. “Structural and practical identifiability analysis of partially observed dynamical models by exploiting the profile likelihood”. In: *Bioinformatics* 25.15 (2009), pp. 1923–1929.

[13] Marisabel Rodriguez Messan et al. “Mathematical model of a personalized neoantigen cancer vaccine and the human immune system”. In: *PLoS computational biology* 17.9 (2021), e1009318.

[14] Wencel Valega-Mackenzie, Jason Bintz, and Suzanne Lenhart. “Resource allocation in a PDE ecosystem model”. In: *Journal of Mathematical Biology* 86.6 (2023), p. 96.

[15] Osman N Yogurtcu et al. “TCPro: an in silico risk assessment tool for biotherapeutic protein immunogenicity”. In: *The AAPS journal* 21.5 (2019), pp. 1–12.
